# Activation of the GABA-alpha receptor by berberine rescues retinal ganglion cells to attenuate experimental diabetic retinopathy

**DOI:** 10.3389/fnmol.2022.930599

**Published:** 2022-08-09

**Authors:** Wangyi Fang, Xiaojing Huang, Kaicheng Wu, Yuan Zong, Jian Yu, Huan Xu, Jiemei Shi, Jiaojiao Wei, Xujiao Zhou, Chunhui Jiang

**Affiliations:** ^1^Department of Ophthalmology and Vision Science, Eye and ENT Hospital, Fudan University, Shanghai, China; ^2^Key Laboratory of Myopia of State Health Ministry, and Key Laboratory of Visual Impairment and Restoration of Shanghai, Shanghai, China; ^3^Department of Ophthalmology, Shanghai Children's Hospital, School of Medicine, Shanghai Jiao Tong University, Shanghai, China; ^4^Department of Ophthalmology, Shanghai Pudong New Area Gongli Hospital, Shanghai, China; ^5^Eye Institute, Eye and ENT Hospital, State Key Laboratory of Medical Neurobiology, Shanghai Medical College, Institutes of Brain Science and Collaborative Innovation Center for Brain Science, Fudan University, Shanghai, China

**Keywords:** diabetic retinopathy, GABA receptor, berberine, retinal ganglion cell, neuroprotection

## Abstract

**Purpose:**

The aim of this study was to investigate the role and mechanism of berberine (BBR) in the protection of injured retinal ganglion cells (RGCs) in diabetic retinopathy (DR).

**Methods:**

Experimental diabetic retinopathy rat model was successfully induced by a single intraperitoneal injection of streptozotocin (STZ, 60 mg/kg) in male SD rats with sufficient food and water for 8 weeks. Animals were randomly divided into four groups: (1) non-diabetic, (2) diabetic, (3) diabetic + BBR + PBS, and (4) diabetic + BBR + SR95531. BBR (100 mg/kg) was given daily by gavage to rats in the group (3) and group (4) for 8 weeks, and weekly intravitreal injections were conducted to rats in the group (3) with 5 μL of 1×PBS and rats in the group (4) with 5 μL of GABA-alpha receptor antagonist SR95531 to investigate the underlying mechanisms. The survival and apoptosis of RGCs were observed by fluorescence gold labeling technology and TUNEL staining. Visual function was evaluated by visual electrophysiological examination. Western blotting and immunofluorescence staining were used to analyze the expression of GABA-alpha receptors in RGCs.

**Results:**

In an animal model, BBR can increase the survival of RGCs, reduce RGCs apoptosis, and significantly improve the visual function. The reduction of GABA, PKC-α, and Bcl-2 protein expression caused by DR can be considerably increased by BBR. SR95531 inhibits BBR's protective effect on RGC and visual function, as well as its upregulation of PKC-α and Bcl-2.

**Conclusion:**

BBR is a promising preventive or adjuvant treatment for DR complications, and its key protective effect may involve the regulation of RGC apoptosis through the GABA-alpha receptor/protein kinase C-alpha (GABAAR/PKC-α) pathway.

## Introduction

Diabetic retinopathy (DR) is one of the most common and severe complications of diabetes mellitus (DM) (Cheung et al., 2010). Berberine (BBR) is a traditional Chinese medicine that can be isolated and purified from *Coptis chinensis* and has had a history of safe clinical use for many years (Kumar et al., 2015; Cicero and Baggioni, 2016). Since the beginning of the 21^st^ century, researchers have found that BBR has preventive and therapeutic effects on diabetic complications, such as renal injury, microvascular injury, and learning and memory impairment, through its antioxidant and anti-inflammatory effects (Zhang et al., 2012; Lin and Zhang, 2018; Ma et al., 2018; Singh et al., 2019; Shinjyo et al., 2020). However, there were few reports on the effect of BBR on retinal complications, especially DR.

DR is thought to result from pathological changes mainly involving retinal microvasculature. However, an increasing number of studies had been focused on neural degeneration in DR (Barber, 2003; van Dijk et al., 2010; Chen et al., 2015; Ng et al., 2016; Potilinski et al., 2020). For example, in diabetic patients, optical coherence tomography (OCT) has revealed thinning of the ganglion cell layer (GCL) and retinal nerve fiber layer (RNFL) in the macular region, which is associated with the course of diabetes (Kim et al., 2018). Also in patients with DR, the change of retinal ganglion cells (RGCs) was detected before the discovery of vascular damage (van Dijk et al., 2010). These indicated that neural degeneration might occur before the damage of blood–retinal barrier (BRB). Among all the neurons, RGC was reported to be most affected, and this might cause a severe decline in visual function in the early course of diabetes (Barber et al., 1998; Ng et al., 2016; Potilinski et al., 2020; Yang et al., 2020).

In the retina, neurotransmitters are directly involved in the regulation of the neuronal microenvironment (Ishikawa et al., 1996; McCall et al., 2002; Shaked et al., 2002). Glutamate-mediated excitatory synaptic transmission and GABA-mediated inhibitory synaptic transmission are important for maintaining the excitatory/inhibitory balance in the nervous system. The balance between them is an important way to prevent the hyperexcitability of neurons, while in DM, retinal ischemia and hypoxia could lead to an increase in excitatory neurotoxicity and neuronal death due to abnormal or excessive release of glutamate (Madl and Royer, 2000). Previous studies found GABAA receptor subunits on nearly all retinal neurons (Sawant et al., 2021), including amacrine and ganglion cells, identified a well-characterized RGC type in the mouse retina: the ON-sustained alpha RGC, and discovered mixed GABA-glycine receptor synapses across this RGC type, revealing the presence of “mixed” inhibitory synapses in the retinal circuit (Popova, 2014). In addition, our previous research has shown that the retinal GABAergic synaptic pathway is implicated in RGC neuroprotection in chronic glaucoma rat models (Zhou et al., 2017a).

The potential neuroprotective impact of BBR on the retina of DR rats, as well as its putative mechanism, will be the subject of this study. Our current research examines whether BBR increases neuronal survival and function, as well as the link between BBR and the GABA receptor, on a cellular and tissue level. Ultimately, we will describe the possible signaling pathways of this neuroprotective effect and put forward the remaining questions in this field of research.

## Materials and methods

The animal experiments were approved by the Institutional Animal Care and Use Committee of Eye and Ears, Nose, and Throat Hospital, Fudan University. All rats were housed in a specific pathogen-free facility with free access to food and water on a 12-h day/night cycle (lights on at 08:00 and off at 20:00).

### Animals

All experiments were performed in adult male Sprague-Dawley rats (200–250 g; 6 weeks old; JSJ Laboratory Animal Co., Ltd., Shanghai, China). The animals were randomly divided into four groups: (1) a non-diabetic group; (2) diabetic group; (3) diabetic + BBR + PBS group; and (4) diabetic + BBR + SR95531 (2-[3-carboxypropyl]-3-amino-6-methoxyphenyl-pyridazinium bromide) group. Diabetes was induced by a single intraperitoneal injection of 60 mg/kg body weight STZ (Sigma, St. Louis, MO). Age-matched non-diabetic animals were injected with an equal volume of citrate buffer (Sakai et al., 1995; Lamuchi-Deli et al., 2017). The induction of diabetes was confirmed by measuring the glucose concentration in blood samples collected from the tail vein using a commercially available blood glucose meter (One Touch Ultra Easy, Johnson & Johnson, USA) with matching glucose test strips. Body weight and blood glucose concentration were measured before the injection and every 2 weeks after. A glucose level of more than 16.7 mmol/L 72 h after the injection of STZ was taken to indicate the successful induction of diabetes. BBR (97%, Macklin Lab Co., Ltd, Shanghai, China) was dissolved in 0.9% saline (100 mg/kg) prior to use. Rats in the group (3) received 100 mg/kg BBR by gavage daily for 8 weeks with 5μL of 1×PBS intravitreal injection weekly after STZ intraperitoneal injection (diabetic + BBR + PBS), and rats in the group (4) received daily 100 mg/kg BBR by gavage with 5μL of SR95531 (100 M, MW = 368.23, 10 mg SR95531 dissolve in 271.5694 ml 1X PBS solution. Please refer to **Figure 3A** for details). For intravitreal injection, the animals were deeply anesthetized by intraperitoneal injection of chloral hydrate (20%, 2 ml/kg). Oxybuprocaine hydrochloride (0.4%; Santen Pharmaceutical Co., Ltd.) was applied as a topical anesthetic, and 0.3% tobramycin (Tobres; Alcon-Couvreur) was applied to prevent postsurgical infection. Animals were sacrificed at 8 weeks by cervical dislocation under deep anesthesia, respectively, after modeling and intervention.

### Patch clamp technology and whole-cell recording

Retinal slice preparation and the voltage clamp recordings of GABAergic mIPSCs of whole RGC cells were described previously (Zhou et al., 2017a,b, 2018). Following decapitation, the eyeballs of rats were removed rapidly and transferred to ice-cold (4 °C) artificial cerebrospinal fluid (ACSF compounds: sodium pyruvate 3, NaHCO3 26, NaH2PO4 1.25, sucrose 124, KCl 3, MgCl2 3.8, CaCl2 0.2, and glucose 10, pH 7.4, in mM) in which sucrose (124 mM) was substituted for NaCl. Before the cells were used in patch-clamp experiments, all sections were incubated in oxygen-saturated ACSF equilibrated with 95% O_2_ and 5% CO_2_. Afterward, the retinal slices were placed in a recording chamber, and the oxygenated ACSF was continuously perfused at a rate of 2~3 ml/min. Interelectrode fluid with Lucifer yellow was injected into the RGC *via* a glass microelectrode, the cell body, and the deformation of the axon, and dendrites of RGCs could be clearly distinguished by the combination of infrared differential interference contrast (IR-DIC) microscopy (Nikon, Tokyo, Japan) with a 40× magnification water-immersion objective lens. Whole-cell frequency and amplitude of mIPSCs were recorded with an intracellular solution containing (in mM) CsCl 150, CaCl_2_ 0.1, MgCl_2_ 1, HEPES 10, EGTA 1, GTP-Na 0.4, and ATP-Mg 4, and Lucifer Yellow 5 (pH 7.2 adjusted with CsOH, 275–290 mOsm/l); the electrode impedance was 4–8 M*Ω*. When the cells were ruptured, whole-cell configuration was attempted in RGC membranes under a glass pipette tip, and the neurons were voltage-clamped at −70 mV using an Axopatch-Multiclamp 700B Amplifier (Molecular Devices, Foster City, CA, USA) (sampling frequency at 10 kHz, filter frequency at 1 kHz) coupled to a digital–analog converter Digidata 1440A system (Axon Instruments, Foster City, CA, USA). The 700B amplifier entirely canceled fast capacitance and partially canceled cell capacitance. The cells were kept at the resting membrane potential for at least 15 min after steady current recording (control) before medicines were given using a gravity-fed superfusion device. In a traditional pharmacological separation experiment, the following medications were delivered using a gravity-fed superfusion system for patch-clamp recordings of miniature inhibitory postsynaptic currents. Tetrodotoxin was used to abolish spontaneous action potentials (TTX, 1 μM); other drugs applied included the ionotropic glutamate receptor antagonists 6-cyano-7-nitroquinoxaline-2, 3-dione (CNQX, 10 μM) and D-2-amino-5-phosphonovalerate (AP5, 50 μM), and the glycine receptor antagonist strychnine (5 μM). The effects of medications on miniature inhibitory postsynaptic currents (mIPSCs) were measured using Clampfit 10.2 (Axon Instruments). The synaptic activity was analyzed using Mini-Analysis (Synaptosoft) and Origin 8.0 software.

### Western blotting

Protein preparation and Western blotting were performed according to previously described methods (Zhou et al., 2017a) with modifications. Briefly, retinal lysates were centrifuged at 12,000 rpm for 10 min at 4 °C. In total, 20 μg of each sample was separated by SDS-PAGE and electrotransferred onto PVDF membranes (Immobilon-P; Millipore). The membranes were blocked with 5% non-fat milk for 1 h at room temperature and then incubated overnight at 4 °C with the following primary antibodies: GABA-alpha receptor (GABAAR; ab33299, rabbit, 1:500; Abcam), protein kinase C-alpha (PKC-α; ab32122, rabbit, 1:1,000; Abcam), and B-cell lymphoma-2 (rabbit, 28 kDa, 1:1,000, Beyotime Institute of Biotechnology, China). The membranes were then incubated with horseradish peroxidase-conjugated AffiniPure Goat Anti-Rabbit IgG (H+L) (1:5,000; Cell Signaling Technology). The relative intensities of the protein bands were quantified by scanning densitometry using ImageJ software. Mouse monoclonal antibodies against β-tubulin (ab78078, 1:2000; Abcam) and β-actin (mouse, 43 kDa, A5441, 1:5,000, Sigma-Aldrich, USA) were used as internal standards.

### Immunofluorescence staining

Briefly, 10-μm-thick cryosections were fixed in 4% paraformaldehyde for 20 min at room temperature and then incubated in 0.1% Triton X-100/PBS for 30 min at 37 °C. After incubation in 3% bovine serum albumin/PBS for 1 h at room temperature, the cryosections were incubated with a rabbit polyclonal antibody against GABAAR (Sigma-Aldrich catalog #A2052, RRID: AB_477652, 1:100) for 1 day at 4 °C to label GABAergic AII amacrine cells. An Alexa Fluor 555-conjugated donkey anti-rabbit IgG antibody (Thermo Fisher Scientific catalog #A-31572, RRID: AB_162543, 1:1,000) was used as the secondary antibody. The sections were counterstained with the nucleic acid stain Hoechst 33258 (Thermo Fisher Scientific catalog #H3569, RRID: AB_2651133, 1:2,000) in PBS. A laser scanning confocal microscope with a 63× oil-immersion objective lens (TCS SP8; Leica Microsystems) was used for imaging.

### TUNEL staining

TUNEL staining was performed on 10-μm-thick cryosections using the *In Situ* Cell Death Detection Kit (#12156792910, Roche, CHE). The TUNEL signal was visualized using a confocal laser scanning microscope with a 40× objective lens. Fluorescence images of TUNEL-positive cells were obtained at an excitation wavelength of 555 nm (red), and the cell nuclei were stained blue with Hoechst 33258.

### Fluorescent gold retrograde labeling of RGCs

Rats from the different groups were anesthetized and injected with the fluorescent tracer 3% FluoroGold (2 μL; Sigma) diluted in saline into the bilateral superior colliculi (6.0 mm posterior and 2.0 mm lateral to bregma and 4–4.5 mm deep) *via* a microsyringe as previously described (Zhou et al., 2017a) 5 days before sacrifice. Five days after FluoroGold injection, the retinas were isolated, dissected, divided into four quadrants, and flat-mounted on glass slides with the GCL facing up. Each quadrant was further divided into central (1.5 mm from the optic disk) and peripheral regions (3 mm from the optic disk). Two fields in each region were randomly selected and counted. Totally 16 microscopic fields in each retina were counted and imaged using a laser scanning confocal microscope (TCS SP8; Leica Microsystems) at a final magnification of 3,200 (scale bar, 50 μm). For each image taken, the number of labeled cells was counted two times by an investigator who was blinded to the grouping, and the average was used as the final result.

### ERG and measurement of oscillatory potentials and the photopic negative response

Eight weeks after the model was established, full-field ERG was performed, and the phNR was evaluated using an Espion Diagnosys System (Diagnosys LLC, Littleton, MA, USA). According to the procedures described (Zhou et al., 2017a), rats were dark-adapted for at least 6 h and prepared under dim red illumination. After the rats were anesthetized, the pupils were dilated, and the corneas were precoated with 2.5% hydroxypropyl-methylcellulose solution (Gonak; Akorn, Lake Forest, IL, USA). ERG was performed with a pair of gold wire loop electrodes that contacted the surface of the cornea. The responses were amplified and bandpass-filtered between 0.3 and 300 Hz, and the luminance range of the light stimuli was 0.003 to 10.0 (P) cd.s/m2. The amplitudes of the a- and b-waves and OPs were measured. After full-field ERG was performed and OPs were recorded, light stimuli were delivered at four different stimulus strengths (11.38 cd.s/m2–0.33 Hz, 11.38 cd.s/m2–1 Hz, 22.76 cd.s/m2–0.33 Hz, and 22.76 cd.s/m2–0.33 Hz) in a four-step test using a ColorDome unit to measure the phNR.

### Statistical analysis

All data are presented as the mean ± SEM. Student's *t*-test was used for comparing between two groups, and an one-way analysis of variance (ANOVA) with Bonferroni's *post-hoc* test was for comparing among multiple groups. In all tests, a *P*-value of < 0.05 was considered statistically significant.

## Results

### The diabetic-induced change in the retinal GABA system was reversed by BBR

**Figure 1:**
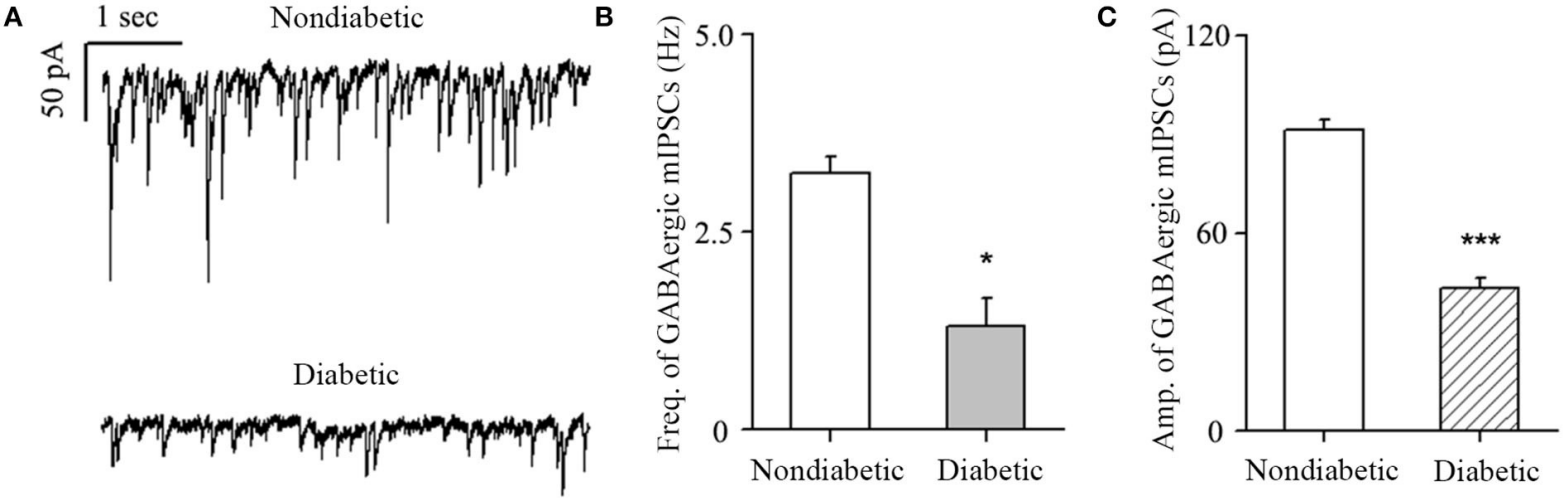
Changes in GABA current frequency and amplitude. **(A)** Representative traces of voltage clamp recordings of GABAergic mIPSCs in the presence of TTX (1 μM) in non-diabetic and diabetic retinas at 4 weeks after diabetic model establishment. The baseline frequency and amplitude of mIPSCs in the retina were markedly reduced in the diabetic group compared with the non-diabetic group. **(B,C)** Histograms showing the mean frequency and amplitude of the mIPSCs for non-diabetic and diabetic retinas. *p < 0.05, ***p < 0.001 (Student's paired *t*-test).

**Figure 2:**
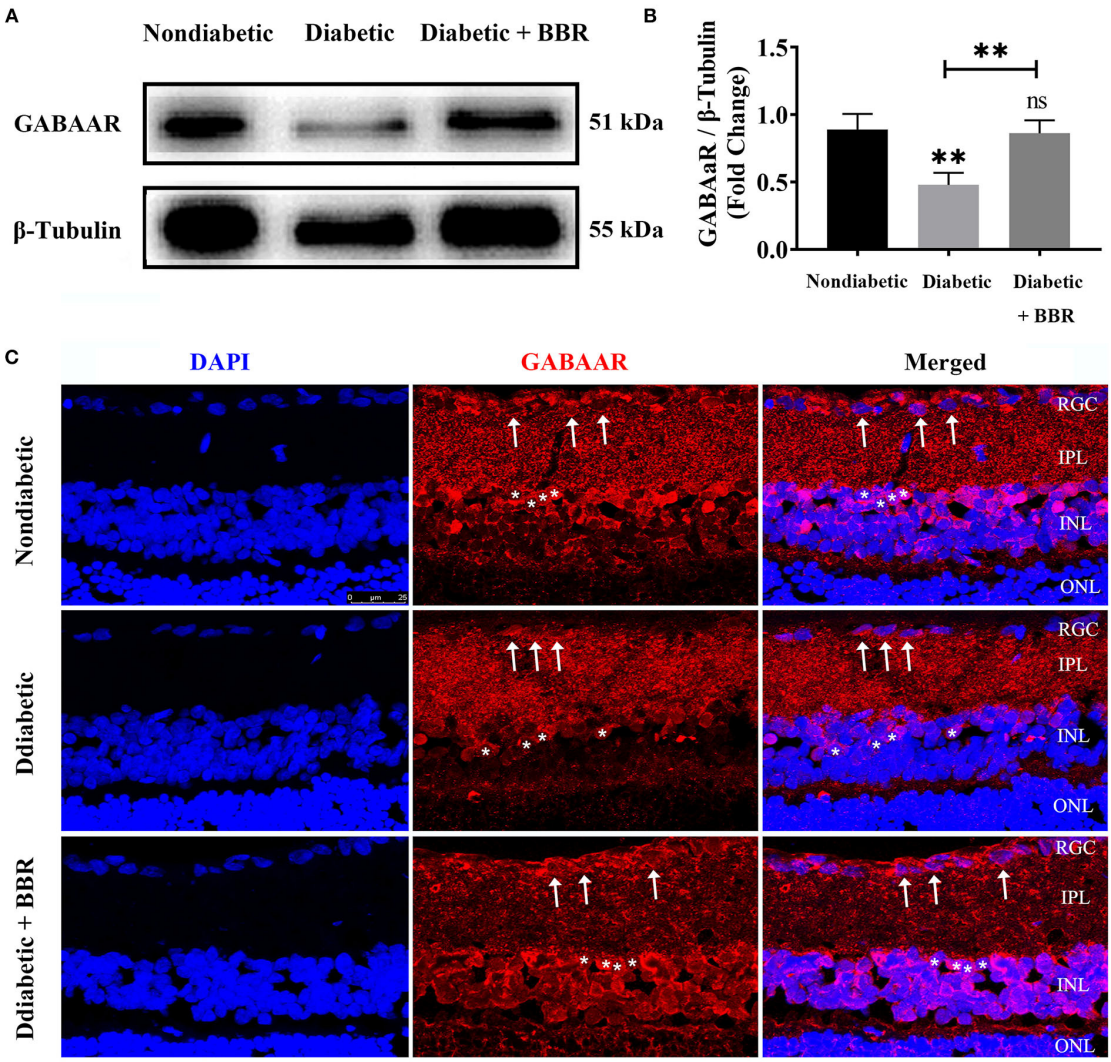
Changes in GABA-alpha receptor expression in the retinas of the three groups. **(A)** Representative Western blots of GABA-alpha receptor expression in retinas of non-diabetic, diabetic, and diabetic + BBR + PBS groups. The expression of the 51 kDa protein was decreased in the diabetic group, and the decrease was reversed in the diabetic + BBR + PBS group with BBR administration. **(B)** Quantitative analysis of the GABA-alpha receptor confirmed the downregulation of GABAAR protein expression in diabetic retinas, and BBR treatment reversed the decrease in GABAAR (ns: no significant difference; **P < 0.01; one-way analysis of variance). **(C)** Representative images of immunofluorescence staining of 10-μm-thick frozen retinal sections from non-diabetic and diabetic rats treated with or without BBR. Fewer ganglion cells with red fluorescence in the retina were observed in the diabetic group (middle panel) than in the non-diabetic group (top panel). More red fluorescence was observed in retinas of the diabetic + BBR + PBS group than the diabetic group (scale bar, 25 μm). The white arrows indicate RGCs; the asterisks indicate amacrine cells. BBR, berberine; GABAAR, GABA-alpha receptor.

The diabetic rat model was successfully established by the single intraperitoneal injection of 60 mg/kg STZ. A diabetic retinal injury was observed and confirmed in the STZ-induced rat model through commonly used laboratory and histological techniques. A significant increase in random blood glucose levels and decrease in weight were detected in the rats in the diabetic group compared with those in the non-diabetic group (Table 1), which is consistent with previous reports (Sakai et al., 1995). GABA is the chief inhibitory neurotransmitter in the central nervous system of vertebrates, where it acts at GABAARs, which play a central role in reducing neuronal excitability. The GABA tonic inhibitory current in RGCs has a more significant and rapid effect on cell potential than other inhibitory currents, which is of great significance for monitoring disease onset. We investigated whether synaptic currents were affected in both the non-diabetic and diabetic groups using patch-clamp recordings. The whole-cell patch-clamp results showed that the frequencies and amplitudes of postsynaptic inhibitory GABAergic synaptic currents recorded in the RGC cell membrane were significantly reduced in the diabetic group compared with the non-diabetic one. The frequency of GABAergic miniature inhibitory postsynaptic currents (mIPSCs) was 3.25 ± 0.21 Hz in normal RGCs and 1.31 ± 0.36 Hz in DR RGCs (*n* = 6, *p* < 0.05; Figures 1A,B). The amplitude of the GABAergic mIPSCs decreased from 91.35 ± 3.15 pA in non-diabetic group to 43.38 ± 3.26 pA in diabetic RGCs (*n* = 6, *p* < 0.001; Figures 1A,C). Therefore, we focused on the GABA system and GABA receptors in the remainder of the study and explored the influence of BBR on the retinal GABA system. After intraperitoneal injection of STZ, rats were intragastrically administered BBR for 8 weeks. Western blotting and immunofluorescence staining showed that the expression of retinal GABAAR was markedly downregulated in diabetic group, whereas oral BBR in diabetic rats increased the expression of retinal GABAAR at the eighth week (Figure 2).

**Table 1:** Body weights and non-fasting blood glucose levels of non-diabetic and diabetic rats.

	**Group**	***N***	**Weight(g)**	***P*** **-value**	**blood glucose level (mmol/L)**	***P*** **-value**
Baseline	Non-diabetic	10	234.5 ± 2.46	0.314	5.89 ± 0.23	0.157
	Diabetic	10	239.5 ± 4.15		5.35 ± 0.29	
8w after STZ injection	Non-diabetic	10	508.30 ± 12.90	0.000	7.29 ± 0.40	0.000
	Diabetic	10	218.80 ± 7.18		30.00 ± 1.65	

The data are presented as the mean ± SE of the results. Measure units: body weight, g; blood glucose level, mmol/L. For specific data of body weight and blood glucose of all groups, see Supplementary Table 1.

### The inhibition in apoptosis and increase in survival of RGC induced by BBR in diabetic rats were antagonized by SR95531

**Figure 3:**
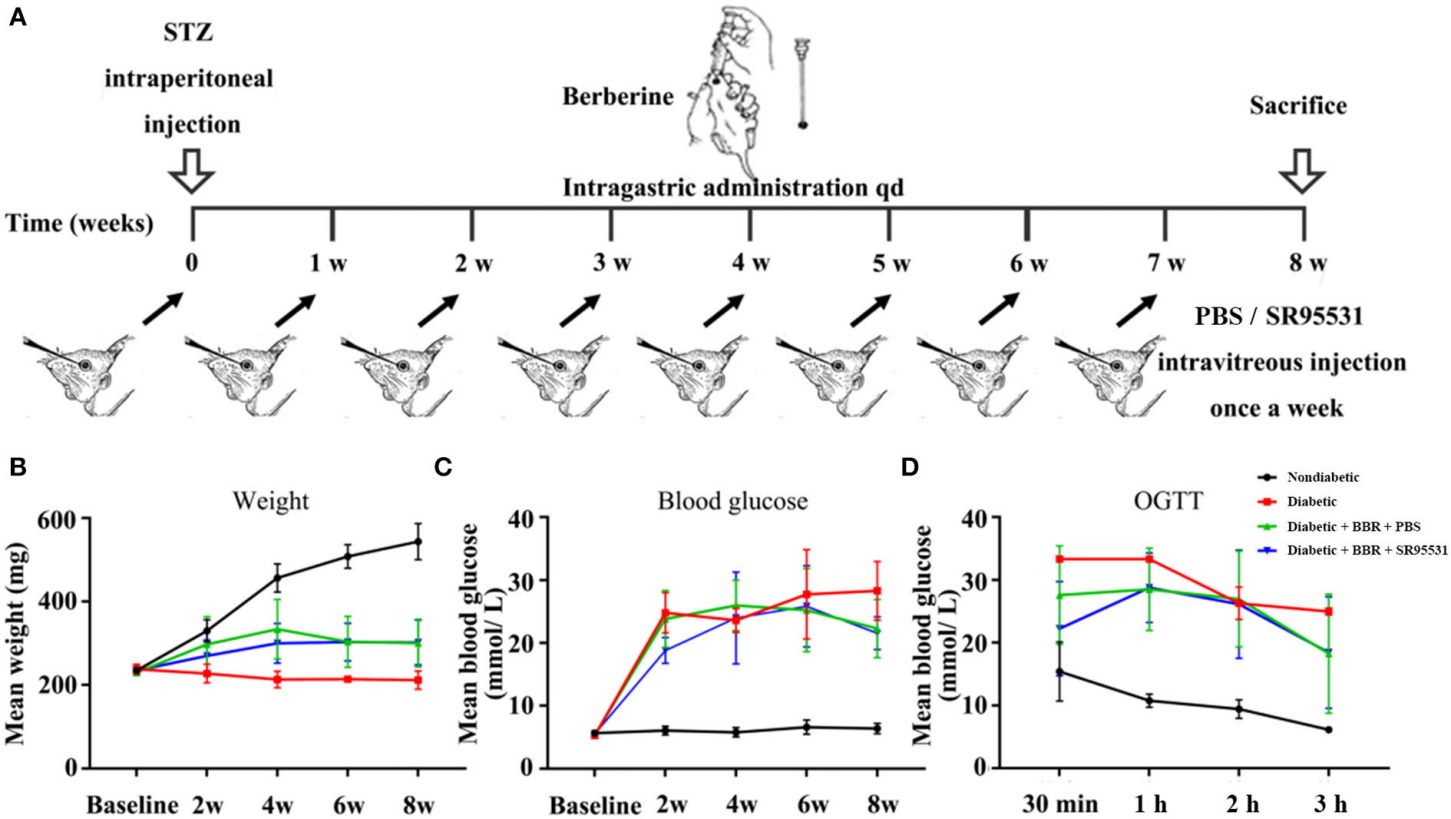
Scheme of model establishment and the conditions of rats in all groups. **(A)** Scheme of experimental diabetic model rat establishment, and BBR and SR95531 administration. **(B)** The body weights of rats in the non-diabetic group, diabetic group, diabetic + BBR + PBS group, and the diabetic + BBR + SR95531 group measured at baseline and at 2 weeks, 4 weeks, 6 weeks, and 8 weeks after STZ injection. **(C)** The blood glucose levels of rats in the non-diabetic group, diabetic group, diabetic + BBR + PBS group, and the diabetic + BBR + SR95531 group measured at baseline and at 2 weeks, 4 weeks, 6 weeks, and 8 weeks after STZ injection. **(D)** Oral glucose tolerance test (OGTT) results of rats in the non-diabetic group, diabetic group, diabetic + BBR + PBS group, and the diabetic + BBR + SR95531 group measured at 8 weeks after STZ injection.

**Figure 4:**
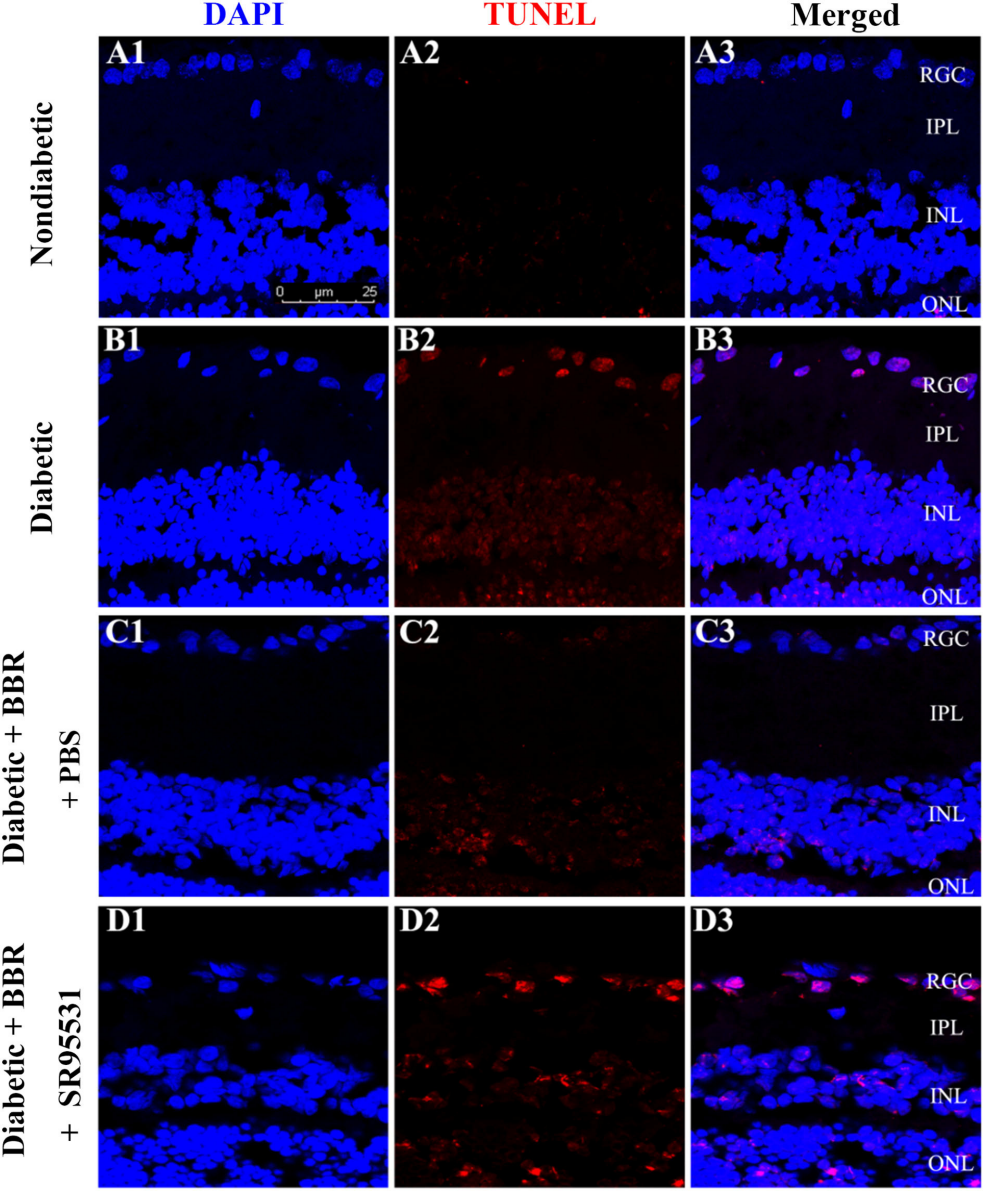
Representative TUNEL (red), DAPI (blue), and merged confocal microscopy images of frozen 10-μm-thick retinal sections from rats in the non-diabetic **(A1–A3)**, diabetic **(B1–B3)**, diabetic + BBR + PBS **(C1–C3)**, and the diabetic + BBR + SR95531 **(D1–D3)** groups. All images were obtained at the same magnification (scale bars, 25 μm; RGC, retinal ganglion cell layer; IPL, inner plexiform layer; INL, inner nuclear layer; ONL, outer nuclear layer).

To elucidate the role of the GABAAR in mediating the protective effect of BBR on RGCs, the selective antagonist SR95531 was injected intravitreally (5 μL, 100 μM) weekly during daily intragastric administration of BBR for 8 weeks (Figure 3A). Four groups in total were established, and weight, blood glucose, and oral glucose tolerance were measured (Figures 3B–D). We performed TUNEL staining to detect apoptotic RGCs in the retinas of all groups. As shown in Figure 4, the TUNEL signal (red) overlapped with the DAPI (blue) signal, demonstrating that cells in multiple layers of the retina, especially the first-layer RGCs, underwent apoptosis. Very few TUNEL-positive cells were observed in the non-diabetic retinas, and the number of TUNEL-positive cells in the retina was significantly lower in the diabetic + BBR + PBS group than in the diabetic + BBR + SR95531 group.

**Figure 5:**
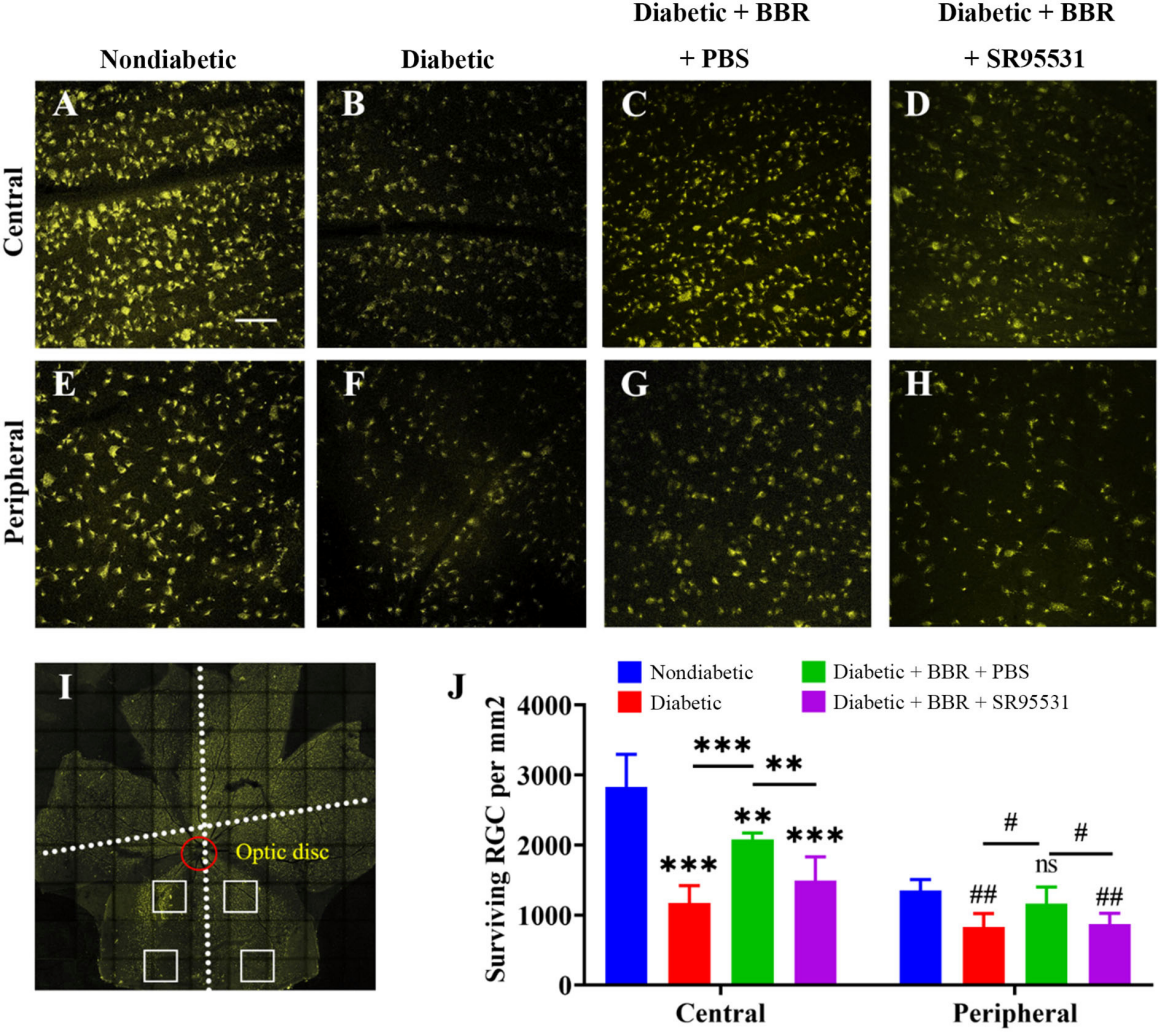
Low-magnification images of FluoroGold-labeled surviving RGCs in the central and peripheral regions of flat-mounted retinas of rats from all four groups. **(A–D)** Fluorescence micrographs of the central regions of flat-mounted retinas from rats of the non-diabetic **(A)**, diabetic **(B)**, diabetic + BBR +PBS **(C)**, and the diabetic + BBR + SR95531 **(D)** groups showing FluoroGold-labeled RGCs. **(E–H)** Fluorescence micrographs of the peripheral regions of flat-mounted retinas from the non-diabetic **(E)**, diabetic **(F)**, diabetic + BBR + PBS **(G)**, and diabetic + BBR + SR95531 **(H)** groups showing FluoroGold-labeled RGCs. **(I)** Flat-mounted retina showing the two eccentric areas of RGC quantification (central and peripheral). Scale bar, 1 mm. **(J)** Quantitative analysis of RGC survival in the four groups, *n* = 5 (ns, no significant difference; ^#^*P* < 0.05; ** and ^*##*^*P* < 0.01; ***P < 0.001; one-way ANOVA; the results are expressed as the mean ± SE. BBR, berberine; RGCs, retinal ganglion cells; scale bar, 50 μm). For specific data, see Supplementary Table 2.

Next, RGC survival was evaluated 8 weeks after the model was established through the fluorescent gold retrograde labeling technique. Representative images of whole flat-mounted retinas (magnification 20 ×) are shown in Figures 5A–I. The density of RGCs (mean ± SE) in the retina was obviously lower in the diabetic group than in the non-diabetic one in both the central (2,829.4 ± 209.0 vs. 1,175.6 ± 110.2 cells/mm^2^, *n* = 5, *P* < 0.001) and peripheral (1,353.0 ± 69.2 vs. 825.2 ± 87.9 cells/mm^2^, *n* = 5, *P* = 0.002) regions. The density of RGCs in the retinas of diabetic + BBR + PBS group was markedly higher than that in the retinas of diabetic group in both the central (2,081.8 ± 41.2 vs. 1,175.6 ± 110.2 cells/mm^2^, *n* = 5, *P* < 0.001) and peripheral (1,163.4 ± 105.6 vs. 825.2 ± 87.9 cells/mm^2^, *n* = 5, *p* = 0.039) regions. The density of RGCs in the eyes of the diabetic + BBR + SR95531 group was similar to that in the diabetic group in both the central (1,492.0 ± 152.7 vs. 1,175.6 ± 110.2 cells/mm^2^, *n* = 5, *P* = 0.131) and peripheral (867.2 ± 71.3 vs. 825.2 ± 87.9 cells/mm^2^, *n* = 5, *P* = 0.720) regions, indicating that SR95531 blocked the effects of BBR on RGCs survival in diabetic retinas (Figures 5B–H,J).

### The beneficial effect of BBR on visual function in diabetic rats was blocked by SR95531

**Figure 6:**
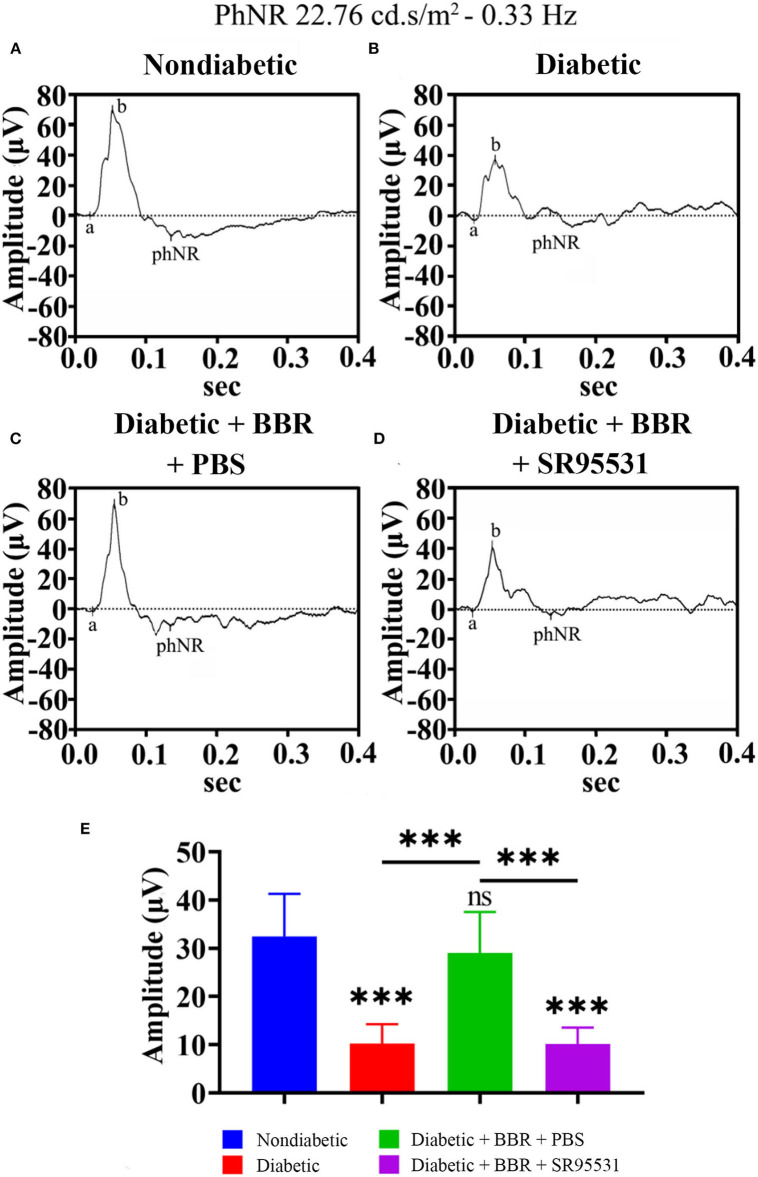
Effect of BBR on retinal function, as determined by the phNR. **(A)** Representative traces of the phNR of a control eye of non-diabetic group at step 3 in the presence of a 22.76-cd.s/m^2^, 0.33-Hz stimulus. **(B)** Representative waves of the eyes of rats in the diabetic group at the same step and in the presence of the same stimulus as in **(A)**. **(C)** Representative waves of the eyes of rats in the diabetic + BBR + PBS group at the same step and in the presence of the same stimulus as in **(A)**. **(D)** Representative waves of the eyes of rats in the diabetic + BBR + SR95531 group at the same step and in the presence of the same stimulus as in **(A)**. **(E)** Quantitative analysis of the phNR amplitude (*n* = 8). The amplitude was normalized to the amplitude of the non-diabetic retinas (mean ± SE; ns, no significant difference compared with the non-diabetic group; ***p < 0.001, one-way ANOVA; phNR, photopic negative response; BBR, berberine).

**Figure 7:**
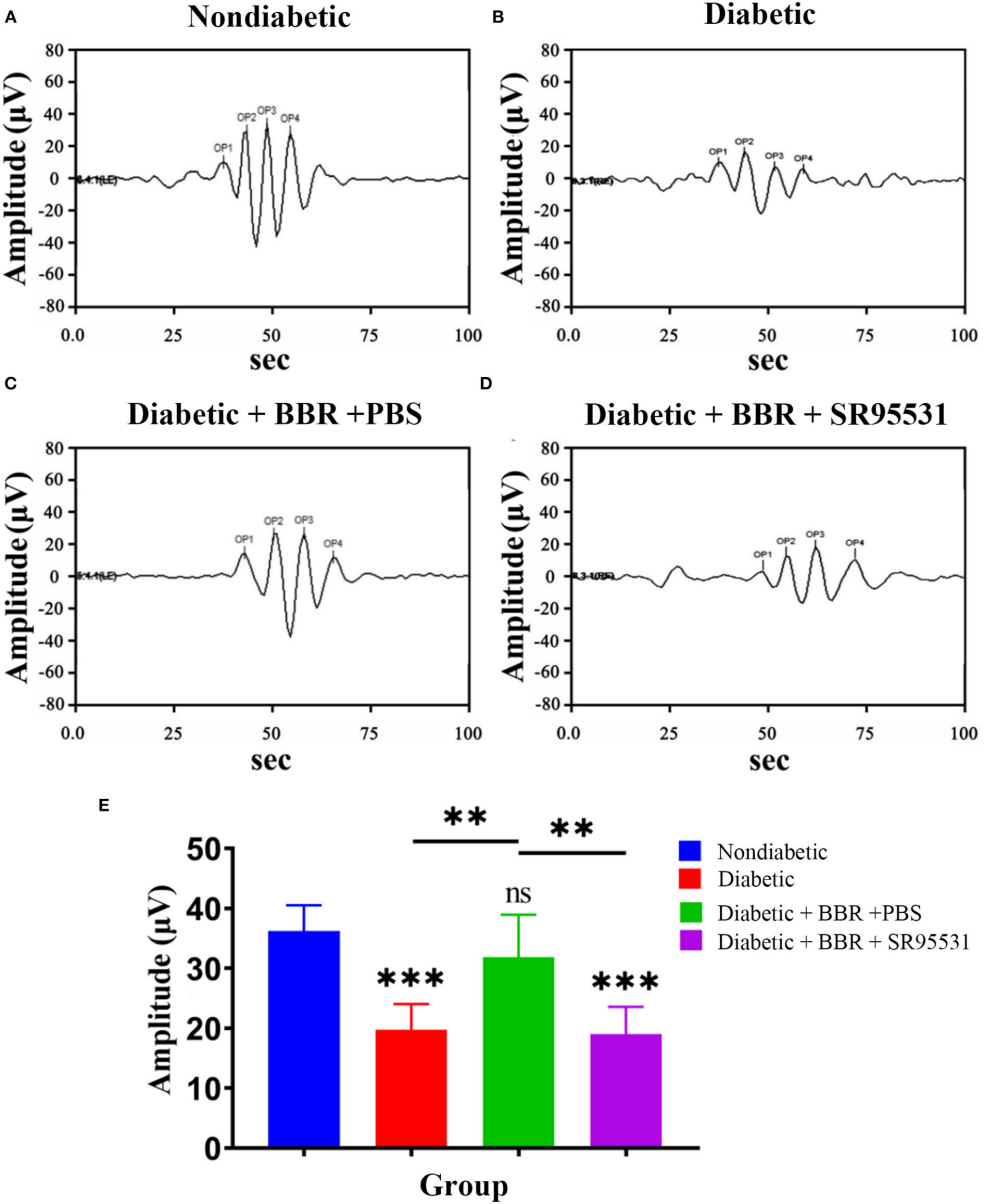
Effect of BBR on retinal function, as determined by OPs. **(A)** Representative traces of the retinal OP waves of a control eye of the non-diabetic group at step 7 in the presence of a dark-adapted 3.0-Hz stimulus. **(B)** Representative waves from the eye of rats in the diabetic group at the same step and in the presence of the same stimulus as in **(A)**. **(C)** Representative waves from the eye of rats in the diabetic + BBR + PBS group at the same step and in the presence of the same stimulus as in **(A)**. **(D)** Representative waves from the eye of rats in the diabetic+ BBR + SR95531 group at the same step and in the presence of the same stimulus as in **(A)**. **(E)** Quantitative analysis of OP amplitude (*n* = 6; ns, no significant difference compared with the non-diabetic group; ^**^P < 0.01; ^***^P < 0.001; one-way ANOVA; OP, oscillatory potential; BBR, berberine).

**Figure 8:**
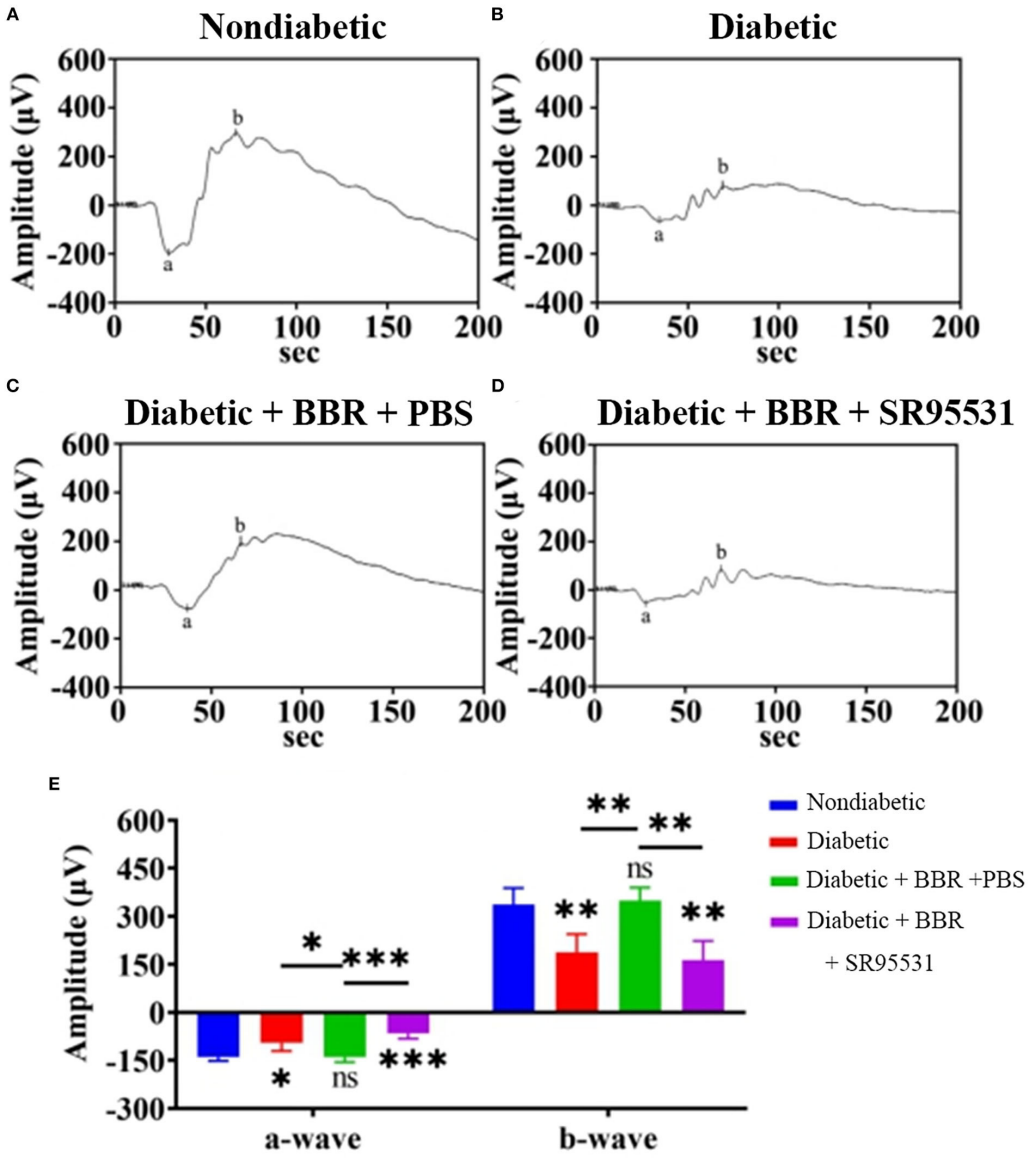
Representative ERG traces in all experimental groups. **(A)** Representative traces of the a-wave and the b-wave of a control eye of the non-diabetic group at step 7 in the presence of a scotopic-adapted 3.0-Hz stimulus. **(B)** Representative waves from the eye of a rat in the diabetic group at the same step and in the presence of the same stimulus as in **(A)**. **(C)** Representative waves from the eye of a rat in the diabetic + BBR + PBS group at the same step and in the presence of the same stimulus as in **(A)**. **(D)** Representative waves from the eye of a rat in the diabetic + BBR + SR95531 group at the same step and in the presence of the same stimulus as in **(A)**. **(E)** Quantitative analysis of a- and b-waves (*n* = 4; ns, no significant difference; ^*^0.01< *P* < 0.05; ^**^P < 0.01; ^***^P < 0.001; one-way ANOVA; BBR, berberine).

In our previous work, we verified that the amplitude of the phNR is positively related to the number of surviving RGCs (Zhou et al., 2017a). Hence, we assessed the functions of both the inner and outer retinal layers by ERG to further evaluate the effect of BBR. The phNR and OP waves were used to assess inner retinal function, especially the function of RGCs, whereas the a- and b-waves were used to evaluate the function of the outer retinal layers. As shown in Figures 6, 7, reductions in the phNR and OPs were observed in rats of the diabetic group compared with the non-diabetic group (phNR: 31.51% ± 4.37%, P_DMvs.CON_ < 0.001; OPs: 54.54% ± 3.43%, *P* < 0.001), and significant increases in the phNR and OPs were observed in rats of the diabetic + BBR + PBS group compared to the diabetic group (phNR: 89.38% ± 9.24%, P_DR+BBRvs.CON_ = 0.7279, P_DR+BBRvs.DR_ < 0.001; OPs: 87.96% ± 3.43%, P_DR+BBRvs.CON_ = 0.4930, P_DR+BBRvs.DR_ = 0.0065). However, the increases in the phNR and OPs were suppressed by SR95531 treatment (phNR: 31.36% ± 3.65%, P_DR+BBR+SR95531vs.CON_ < 0.001; OPs: 52.64% ± 5.55%, P_DR+BBR+SR95531vs.CON_ < 0.001). Similarly, scotopic and photopic ERG responses were significantly decreased in the diabetic group (Figure 8, Table 2), whereas the average amplitudes of a- and b-waves in rats of the diabetic + BBR + PBS group were close to those of the non-diabetic ones (Figure 8, Table 2). SR95531 injection also alleviated the effects of BBR on the ERG response, as no significant differences in ERG a- and b-waves were observed between the diabetic + BBR + SR95531 group and the diabetic group. These results illustrate that the protective effects of BBR were associated with the number of TUNEL-positive cells, RGC density, and RGC function in diabetic eyes, which are attributable to the activation of GABAAR. The blockade of GABAAR in diabetic retinas abolishes BBR-induced neuroprotection.

**Table 2:** Mean amplitude of ERG components in all experimental groups.

**Group**	**PhNR**	**OP**	***a*** **-wave**	***b*** **-wave**
Non-diabetic	32.49 ± 3.097	36.21 ± 1.758	139.9 ± 6.016	337.1 ± 25.71
Diabetic	10.24 ± 1.420^†^,^‡^	19.75 ± 1.900^†^,^‡^	95.39 ± 12.73^†^,^‡^	188.2 ± 28.22^†^,^‡^
Diabetic + BBR + PBS	29.04 ± 3.001	31.85 ± 2.886	139.6 ± 7.750	348.5 ± 20.67
Diabetic + BBR + SR95531	10.19 ± 1.185^†^,^‡^	19.06 ± 2.011^†^,^‡^	65.51 ± 8.438^†^,^‡^	163.9 ± 29.87^†^,^‡^

The data are presented as the mean ± SE of the results. Measure unit: wave amplitude, μV.

† P < 0.05 compared to the controls.

‡ P < 0.05 compared to the diabetic + BBR + PBS group.

(phNR, photopic negative response; OP, oscillatory potential; BBR, berberine).

### BBR might exert its effects through GABAAR/ PKC-α pathway

**Figure 9:**
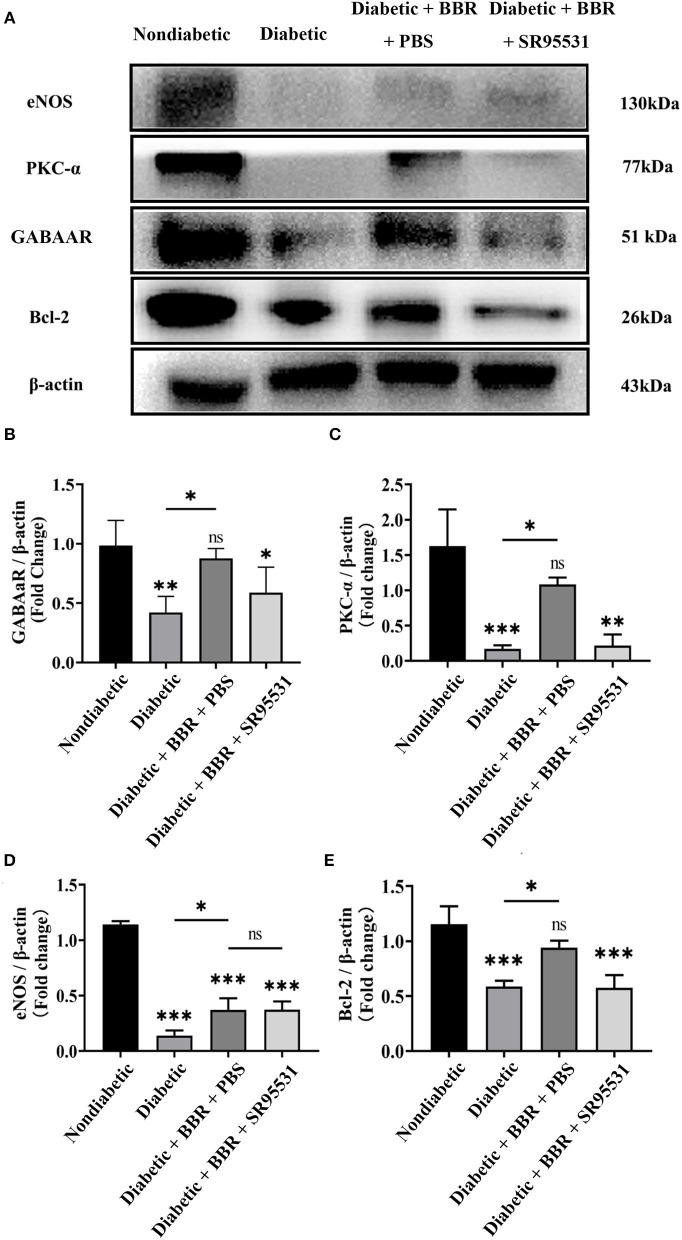
Berberine inhibits RGC apoptosis through the GABAAR/PKC-α pathway. **(A)** Representative Western blots of GABAAR, eNOS, PKC-α, Bcl-2, and β-actin protein expression in different groups. **(B–E)** Statistical analysis of the fold change of GABAAR, eNOS, PKC-α, and Bcl-2 protein expression relative to β-actin. (ns, no significant difference; ^*^P < 0.05, ^**^P < 0.01 and ^***^P < 0.001 vs. the non-diabetic group; one-way analysis of variance).

BBR has been shown to have a regulatory effect on eNOS activity and boost NO production in previous studies. However, our findings revealed that the BBR had no effect on the level of eNOS in the retina (Figures 9A,D). Finally, we investigated the possible signaling pathway underlying the protective effect of BBR on RGCs in the experimental diabetic rats. Western blotting suggested that the expression of GABAAR, PKC-α, and Bcl-2 was greatly reduced in the diabetic group and that BBR significantly restored this reduction. On the contrary, intravitreal injection of the GABAAR-specific blocker SR95531 completely blocked the effect of BBR on PKC-α and Bcl-2 (Figures 9A–C,E). Moreover, the results showed that expression of the PKC-α and Bcl-2 proteins was positively correlated with GABAAR expression and that the protective effects of BBR could be regulated through activation of the GABAAR and downstream PKC-α-mediated signaling pathway.

## Discussion

Diabetic retinopathy (DR)—a common complication of diabetic mellitus (DM), was reported to be found in over 50% of patients with 10 years or longer DM (Song et al., 2018). Previous studies of DR mainly focused on the pathological changes of retinal microvasculature, while recently it was proposed that the DM might also directly compromise the neurons of the retina (Chen et al., 2018; Zhai et al., 2020). Neurodegeneration in the diabetic retina is indicated by RGC loss and thinning of the retinas (Barber and Baccouche, 2017; Eggers and Carreon, 2020; Jadeja and Martin, 2021), and both human and animal studies suggested that changes in retinal neurons occur early after the onset of diabetes and precede the changes of the microvasculature (Barber et al., 1998; Chen et al., 2015; Ng et al., 2016).

Berberine is a kind of Rhizoma Coptidis extract with great clinical application such as in cholecystitis, malaria, diarrhea, and gastrointestinal diseases (Shamsa et al., 1999). Recently, in both *in vivo* studies using human retinal Müller cells and *in vitro* studies using rats, it was found that BBR could protect retinal Müller cells from damage under diabetic conditions. Chen et al. (2018) further reported that in diabetic rat, BBR attenuates the apoptosis of retinal Müller cells through AMPK/mTOR signaling, and Zhai et al. (2020) reported that BBR might inhibit retinal Müller cells' apoptosis *via* the deactivation of the NF-kappa B in both *in vitro* and *in vivo* diabetic model.

In this study, a significant decrease in body weight and increase in blood glucose levels were observed after the injection of STZ, confirming the establishment of DM. In the retina, increasing of TUNEL-positive cells in the RGC layer and decreasing of the amplitude of phNR, OPs, and a- and b- waves of ERG, which were in accordance with early reports (Hancock and Kraft, 2004; Kizawa et al., 2006), were recorded. With the administration of BBR, all these structural and functional impairments mentioned above were reversed, and these strongly suggested a neural protective effect of BBR in diabetic conditions.

The mechanism behind was then studied. Former studies had found that in the early stage of DR, retinal impairment was closely related to the decreased releasing of inhibitory transmitters and increase in excitatory ones, which manifested as an increase of glutamate and a decrease of GABA. While the earlier change of phNR in patients with diabetes reflects reduced input to the retinal ganglion cell from the distal retina, the changes of OPs and phNR found this time were consistent with the results of previous studies (Kizawa et al., 2006) which also suggested the significantly decreased excitability of RGCs. On the contrary, one of the major inhibitory inputs in the retina is mediated by GABAARs (Grunert, 2000), which are expressed on all types of neurons in the vertebrate retina and are the main mediators of miniature inhibitory postsynaptic currents (mIPSCs) in RGCs. Okumichi et al. reported that by blocking the GABA A receptors expressed on RGCs (Okumichi et al., 2008), the death of RGCs induced by oxidative stress was accelerated (Kizawa et al., 2006). Furthermore, in diabetic conditions, the activity and expression of the GABA receptor in the retina were also found to decrease. Based on the findings above, we explored the possible involvement of the GABA pathways. The expression of GABAARs in the retinas was significantly decreased in the DR group as well as the structural and functional damage of RGC. Moreover, with the administration of BBR, the decreasing of GABAARs was revised together with the protection of RGC, while SR95531, a selective competitive antagonist of GABAARs, blocked the protective effect of BBR. These suggested the involvement of the GABAARs pathway in the neuroprotective role of BBR.

Recently, Zan et al. reported that BBR protected diabetic neuropathy through the PKC pathway (Zan et al., 2017), which has a crucial interaction with GABAARs. Previous studies have highlighted the role of PKC in diabetic complications, especially in vascular complications, which in neuropathy-related issues still remain to be further explored. The classical hypothesis is that hyperglycemia activates the DAG-PKC pathway and activation of PKC isoforms causes vascular dysfunction, including retinal vascular dysfunction. Moreover, PKC-β is found to play a key role in VEGF-induced retinal EC permeability by altering occluding phosphorylation. The PKC-β inhibitor was studied both in *in vivo* and *in vitro* experiments, and even clinical trials have shown potential for PKC-β inhibitor as a treatment for diabetic vascular complications (Geraldes and King, 2010). While, Okon et al. (2003) found that PKC inhibition had no effect on a mouse model of diabetes, and there is also research that reported diabetes does not affect nerve PKC activity (Cameron and Cotter, 2002). PKC's multifunctionality is possibly reflected in the debate surrounding its function. In contrast to earlier investigations, our findings indicated that PKC-α was significantly reduced in the diabetic group. For example, Inoguchi T. et. al reported that hyperglycemia activates PKC isoforms in retinal tissues, including PKC-α, -β-δ, and -ε (Inoguchi et al., 1992). The reasons for the discrepancy are unclear, but we hypothesize that they may be related to the different species or duration of experimental DM. The STZ-induced diabetic BB and Sprague-Dawley rats used in the Inoguchi T.'s study persisted for up to 5 weeks, while the rats used in our study persisted for up to 8 weeks. Recent research has shown that diabetes has a specific impact on the expression and subcellular distribution of PKC isozymes in the liver, retina, and cardiovascular tissues of experimentally diabetic rats (Roberts and McLean, 1997). PKC is a family of at least 12 isozymes (Dekker and Parker, 1994), which have distinct activities and are probably controlled in various ways, as evidenced by the fact that they differ not only in their cellular and subcellular distributions, but also in their ability to phosphorylate certain substrates (Sheu et al., 1990). Under our experimental conditions, it was further found that berberine plays a retinal neuroprotective role by restoring the PKC-α reduction in diabetic rats.

On the contrary, PKC-α regulates the level of Bcl-2—a strong anti-apoptotic molecule (Ruvolo et al., 1998; Jiffar et al., 2004; Phatak et al., 2016). This time, in diabetic group, the level of Bcl-2 was significantly decreased, which was restored by BBR, and SR95531 also completely blocked the effect of BBR. Formerly, it was reported that the anti-apoptosis effect of BBR in Parkinson's disease (PD) and Alzheimer's disease (AD) might take place through the restoration of Bcl-2/Bax and Bcl-xl/Bax (Ma et al., 2017), and our findings were in accordance with theirs.

Besides, there is evidence suggesting that eNOS is involved in the pathogenesis of DR (Li et al., 2010; Ma et al., 2014). Previous studies showed BBR can exert regulatory effect on eNOS activity and increase the synthesis of NO. Therefore, the expression of eNOS was examined to explore whether eNOS is involved in the neuroprotective effect of BBR, but similar to the findings of Vorwerk et al. (1997), our result suggested that the BBR did not change the level of eNOS in the retina under diabetic conditions.

The findings suggested the potential neuroprotective effect of BBR. Also, BBR exists in a variety of natural plants and is easy to be extracted. It also was low cost, and had a long history of safe clinical application (Sandeep and Nandini, 2017; Ju et al., 2018; Imenshahidi and Hosseinzadeh, 2019; Suadoni and Atherton, 2021). In addition, BBR can penetrate the BBB successfully (Wang et al., 2005, 2020; Simoes Pires et al., 2014; Abdel Moneim, 2015; Zhou et al., 2016) and may pass through the blood–retinal barrier which has similar structures.

Interestingly, in our results, the a- and b-waves, which reflected the outer retinal function, were also protected by BBR, and this effect and the mechanism will need to be investigated in the future. On the cellular and tissue level, this research revealed whether berberine promotes the neuronal survival and function as well as the relationship with GABA receptor. As far as we know, clinical trials investigating the efficacy of BBR as a diabetes treatment have been conducted. A meta-analysis of these randomized clinical trials indicates that berberine can improve obesity and hyperlipidemia by reducing triglyceride (TG), total cholesterol (TC), and low-density lipoprotein (LDL) and increasing high-density lipoprotein (HDL); has comparable therapeutic effect on type 2 DM through reducing insulin resistance (Harrison et al., 2021); and prevents diabetic encephalopathy and hypertension with no serious side effect (Lan et al., 2015; Ye et al., 2021). Some researchers have suggested that BBR can be used as a potential clinical treatment for diabetic renal fibrosis and diabetic tendon injury. Li et al. reported that BBR may inhibit fibrosis and ameliorate the symptoms of diabetic nephropathy (Li and Zhang, 2017) and Zhu et al. demonstrated that BBR treatment significantly increased autophagy activation and decreased cell apoptosis in tendon tissues of T2DM rats through TUNEL assay and immunohistochemical analysis (Zhu et al., 2022). Based on these findings, we can reasonably predict that BBR could be a promising preventive or adjuvant treatment for DR complications in the clinical prevention and treatment of diabetic retinopathy. To expand our knowledge in this sector, more research should be undertaken.

## Conclusion

BBR can effectively protect against the damage of RGC in rodent diabetic retinas. The protective effect might be related to the activation of GABAARs/PKC-α pathways.

## Data availability statement

The original contributions presented in the study are included in the article/Supplementary material, further inquiries can be directed to the corresponding authors.

## Ethics statement

The animal study was reviewed and approved by the Institutional Animal Care and Use Committee of Eye and Ears, Nose, and Throat Hospital, Fudan university.

## Author contributions

WF and XH designed the experiments, did the experiments, and wrote the manuscript. KW, HX, JS, and JW helped in the experiments. YZ and JY analyzed the data. CJ and XZ modified the manuscript. All authors contributed to the article and approved the submitted version.

## Funding

This project was supported by research grants from the Special Fund for Livelihood Research of Shanghai Pudong New Area Science and Technology Development Fund (PKJ2020-Y27), the National Key Research and Development Plan (2017YFC0108200 and 2017YFC0108201), the Shanghai Natural Science Foundation (19ZR1408400), and the Shanghai Committee of Science and Technology (19441900900).

## Conflict of interest

The authors declare that the research was conducted in the absence of any commercial or financial relationships that could be construed as a potential conflict of interest.

## Publisher's note

All claims expressed in this article are solely those of the authors and do not necessarily represent those of their affiliated organizations, or those of the publisher, the editors and the reviewers. Any product that may be evaluated in this article, or claim that may be made by its manufacturer, is not guaranteed or endorsed by the publisher.
