# Supplementary material for: Activation of the GABA-alpha receptor by berberine rescues retinal ganglion cells to attenuate experimental diabetic retinopathy
Source: Front Mol Neurosci. 2022 Aug 9;15:930599. doi: 10.3389/fnmol.2022.930599 (PMC9396352; doi:10.3389/fnmol.2022.930599)
Supplement: Supplementary file 1 [file Presentation_1.PPTX]

## Slide 1
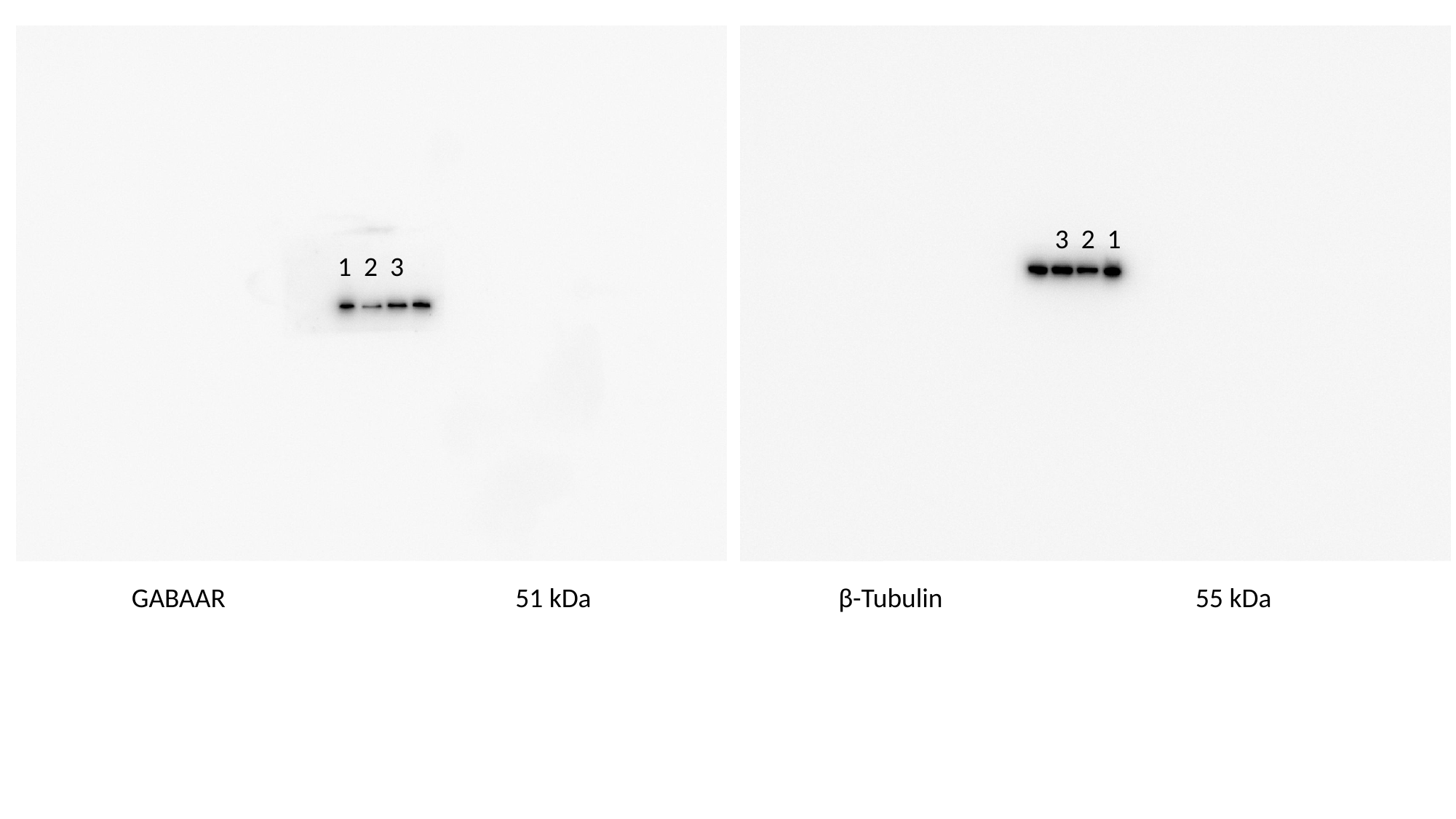

3 2 1
1 2 3
GABAAR 51 kDa
β-Tubulin 55 kDa

## Slide 2
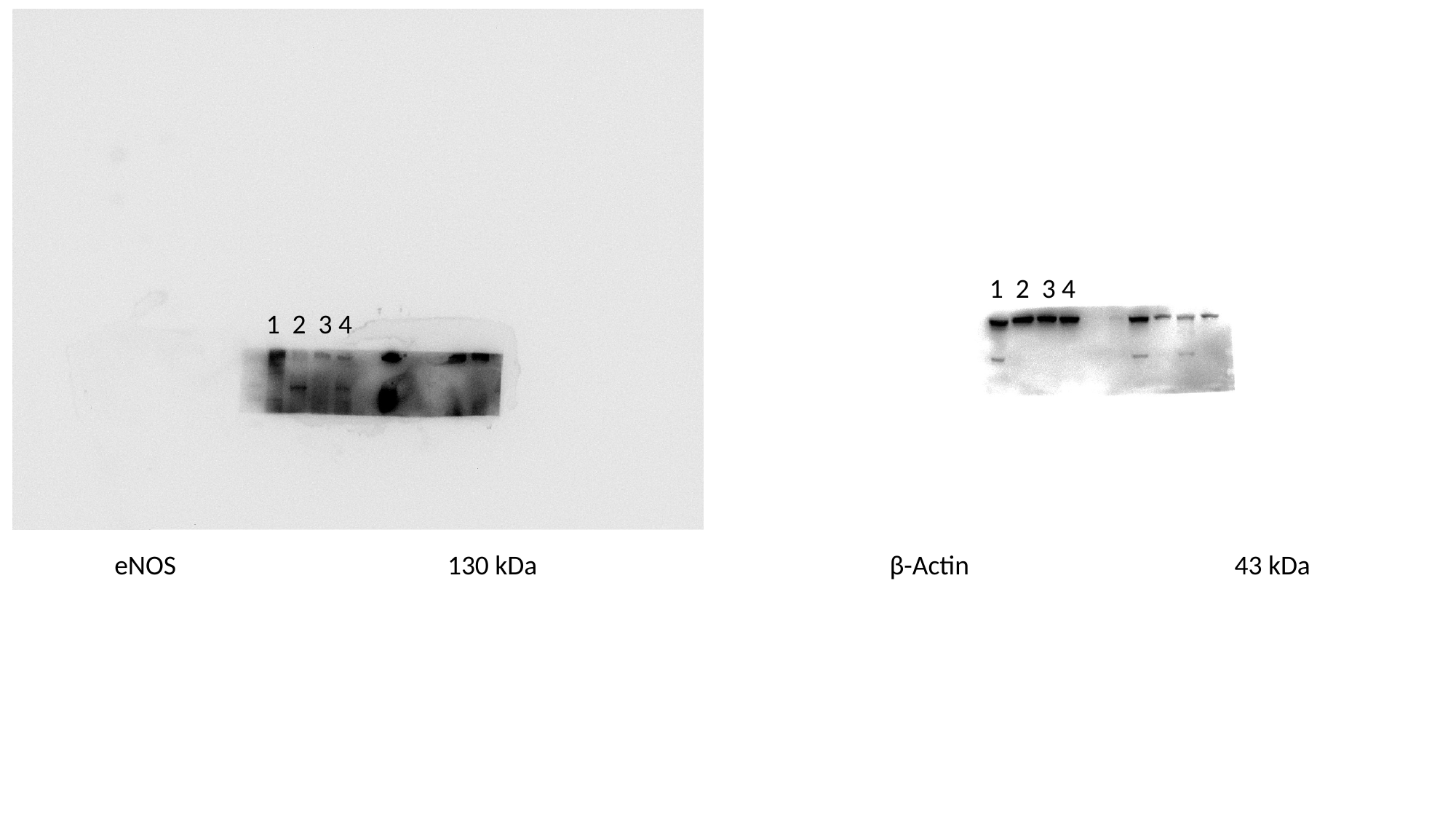

1 2 3 4
1 2 3 4
eNOS 130 kDa
β-Actin 43 kDa

## Slide 3
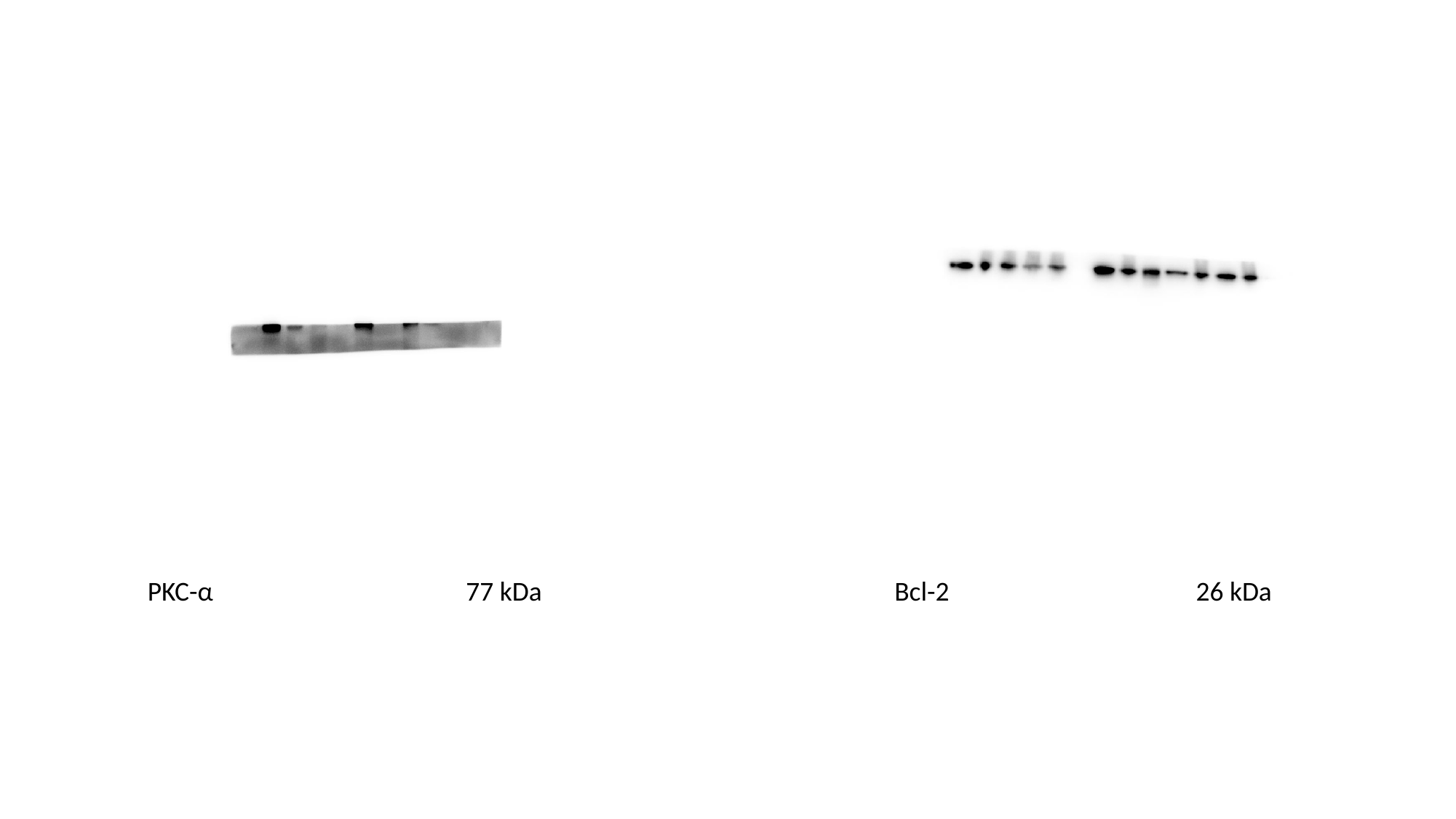

PKC-α 77 kDa
Bcl-2 26 kDa

## Slide 4
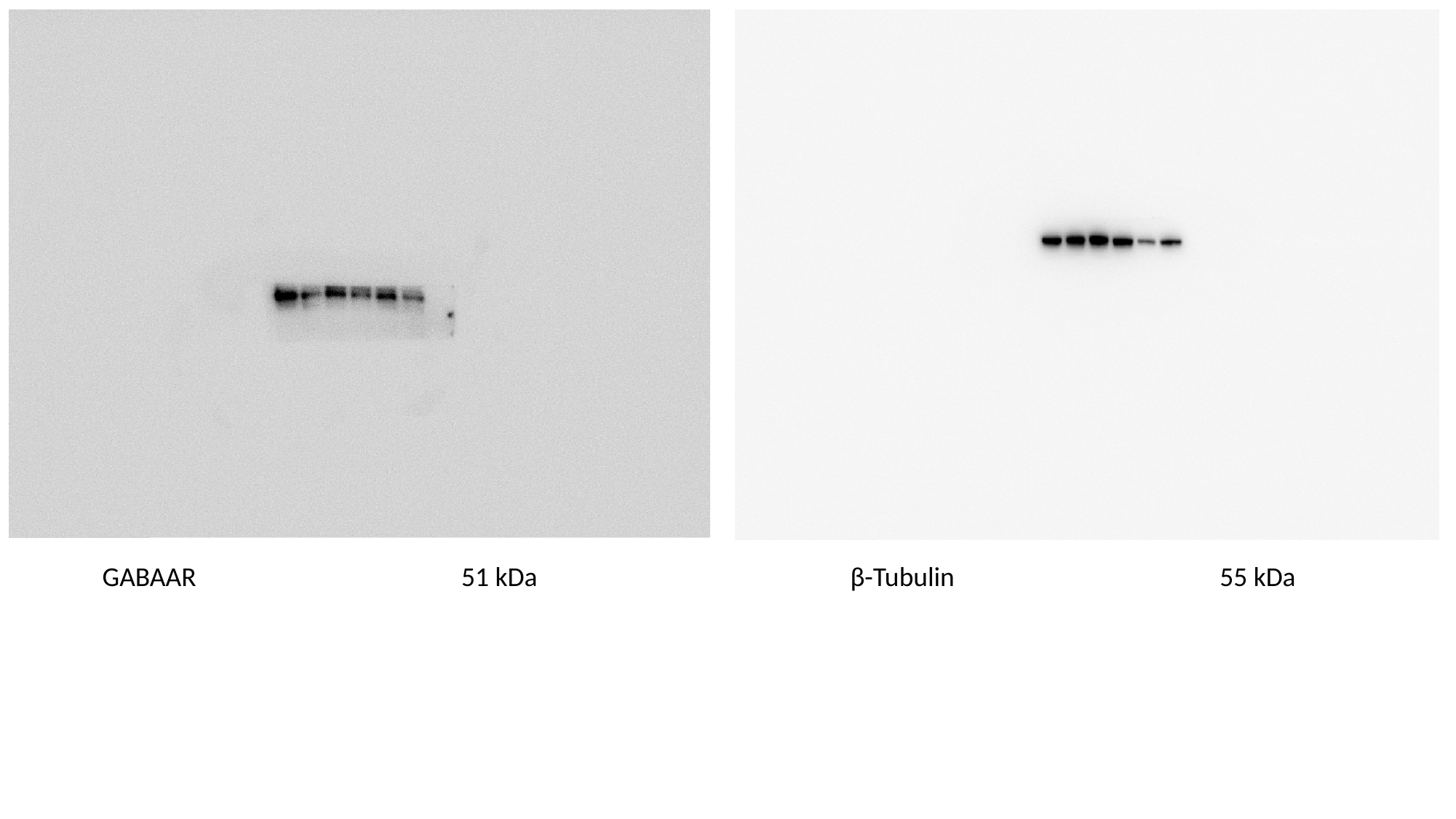

GABAAR 51 kDa
β-Tubulin 55 kDa
