# Supplementary material for: Activation of the GABA-alpha receptor by berberine rescues retinal ganglion cells to attenuate experimental diabetic retinopathy
Source: Front Mol Neurosci. 2022 Aug 9;15:930599. doi: 10.3389/fnmol.2022.930599 (PMC9396352; doi:10.3389/fnmol.2022.930599)
Supplement: Supplementary file 2 [file Data_Sheet_1.docx]

**Supplementary Material**

| **Supplementary Table 1. Body weights and blood glucose levels of the nondiabetic and diabetic rats** | | | | | |
| --- | --- | --- | --- | --- | --- |
| Group | | Nondiabetic | Diabetic | Diabetic + BBR + PBS | Diabetic + BBR + SR95531 |
| Body weights | Baseline | 234.5±2.46 | 237.9±3.73 | 230.1±2.32 | 234.5±2.14 |
|  | 8 w | 543.6±13.66 | 211.80±6.84^†^ | 300.3±18.72^†, ‡^ | 301.9±20.28^†, ‡^ |
| Blood glucose levels | Baseline | 5.65±0.13 | 5.28±0.21 | 5.38±0.18 | 5.53±0.30 |
|  | 8 w | 6.37±0.41 | 28.28±2.33^†^ | 22.28±2.31^†^ | 21.53±1.50^†^ |
| OGTT | 30 min | 15.4±2.10 | 33.3±0.00^†^ | 27.58±3.92^†^ | 22.23±3.75^†^ |
|  | 1 hr | 10.76±0.46 | 33.3±0.00^†^ | 28.5±3.28^†^ | 28.75±2.76^†^ |
|  | 2 hr | 9.42±0.66 | 26.28±1.29^†^ | 26.95±3.82^†^ | 26.13±4.31^†^ |
|  | 3 hr | 6.14±0.21 | 24.95±0.27^†^ | 18.25±4.73^†^ | 18.43±4.44^†^ |

*The data are presented as the mean ± SE of the results.

Measure Unit: body weights, g; blood glucose levels, mmol/L.

^†^ P < 0.05 compared to the nondiabetic group.

^‡^ P < 0.05 compared to the diabetic group.

| **Supplementary Table 2. Density of retinal ganglion cells ( cells/mm2) in four groups of rats.** | | | | |
| --- | --- | --- | --- | --- |
| Group | Nondiabetic | Diabetic | Diabetic + BBR + PBS | Diabetic+BBR+SR95531 |
| Central region | 2829.4 ± 209.0 | 1175.6 ± 110.2 | 2081.8 ± 41.2 | 1492.0 ± 152.7 |
| Peripheral region | 1353.0 ± 69.2 | 825.2 ± 87.9 | 1163.4 ± 105.6 | 867.2 ± 71.3 |

*N = 5, the results are analyzed using one-way ANOVA and expressed as the mean ± SE.
