# Supplementary material for: Activation of the GABA-alpha receptor by berberine rescues retinal ganglion cells to attenuate experimental diabetic retinopathy
Source: Front Mol Neurosci. 2022 Aug 9;15:930599. doi: 10.3389/fnmol.2022.930599 (PMC9396352; doi:10.3389/fnmol.2022.930599)
Supplement: Supplementary file 3 [file Data_Sheet_2.PDF]

Supplementary Material

Activation of the GABA-alpha Receptor by Berberine Rescues Retinal Ganglion Cells to Attenuate Experimental Diabetic Retinopathy

Wangyi Fang[1,2]†, Xiaojing Huang[3]†, Kaicheng Wu[1,2], Yuan Zong[1,2], Jian Yu[1,2], Huan Xu[1,2], Jiemei Shi[1,2], Jiaojiao Wei[1,2], Xujiao Zhou[1,2,4]†*, Chunhui Jiang[1,2]†*

[1] Department of Ophthalmology and Vision Science, Eye and ENT Hospital, Fudan University, Shanghai, 200031, People's Republic of China

[2] Key Laboratory of Myopia of State Health Ministry, and Key Laboratory of Visual Impairment and Restoration of Shanghai, Shanghai, 200031, People's Republic of China

[3] Department of Ophthalmology, Shanghai Pudong New Area Gongli Hospital, Shanghai 200135, People's Republic of China

[4] Eye Institute, Eye and ENT Hospital, State Key Laboratory of Medical Neurobiology, Institutes of Brain Science and Collaborative Innovation Center for Brain Science, Shanghai Medical College, Fudan University, Shanghai, China

†These authors have contributed equally to this work and share first authorship.

***Correspondence**: 1.Chunhui Jiang*, e-mail: chhjiang70@163.com Department of Ophthalmology and Vision Science, Eye and ENT Hospital, Fudan University, 83 Fenyang Rd, Shanghai 200031, People's Republic of China

2. Xujiao Zhou*, e-mail: xujiaozhou@126.com Eye Institute, Eye and ENT Hospital, State Key Laboratory of Medical Neurobiology, Institutes of Brain Science and Collaborative Innovation Center for Brain Science, Shanghai Medical College, Fudan University, Shanghai, China

†* These authors have contributed equally to this work and share Corresponding authorship.

Supplementary Figure

A

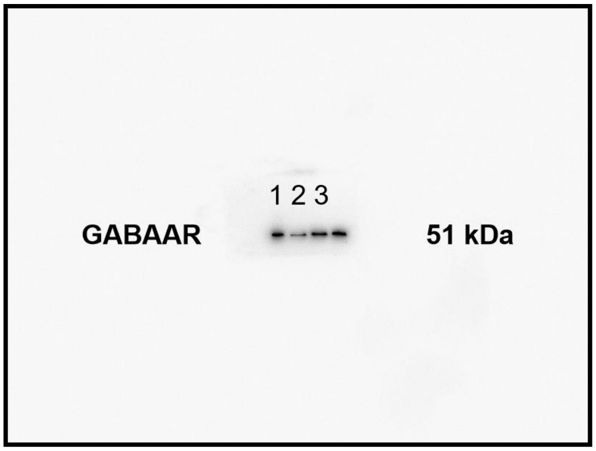

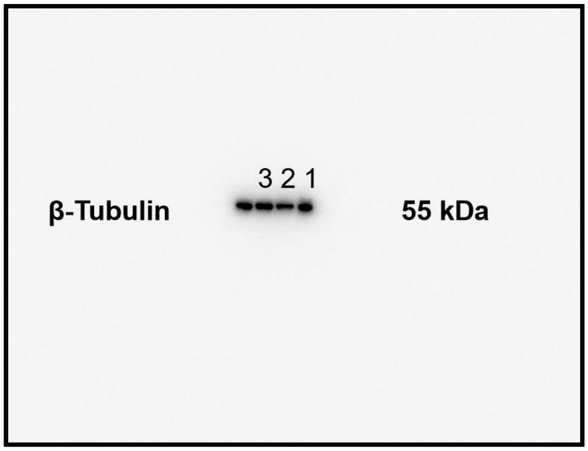

1 - Nondiabetic
2 - Diabetic
3 - Diabetic + BBR

B

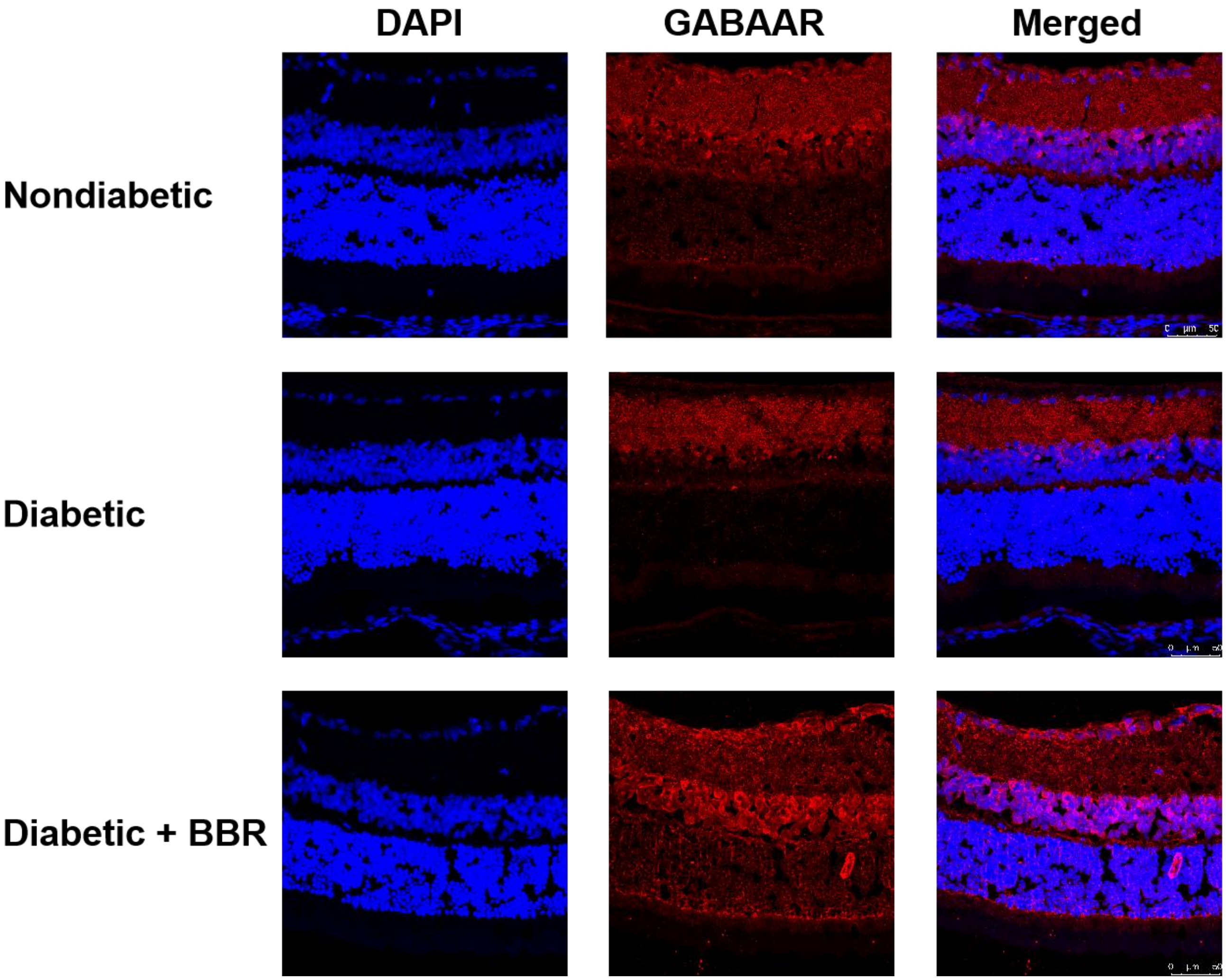

Supplementary Figure S1. Full-length blots of **Figure 2A**, and original images of immnoflorescence staining of **Figure 2C**. **(A)** Western blotting analysis of protein expression in retinas of nondiabetic, diabetic and diabetic + BBR group. Full-length blots of Figure 3A, showing the expression of GABAAR and β-tubulin. **(B)** Original images of Figure 2C taken under laser confocal microscopy after immunoflorescence staining. (scale bar, 50 μm)

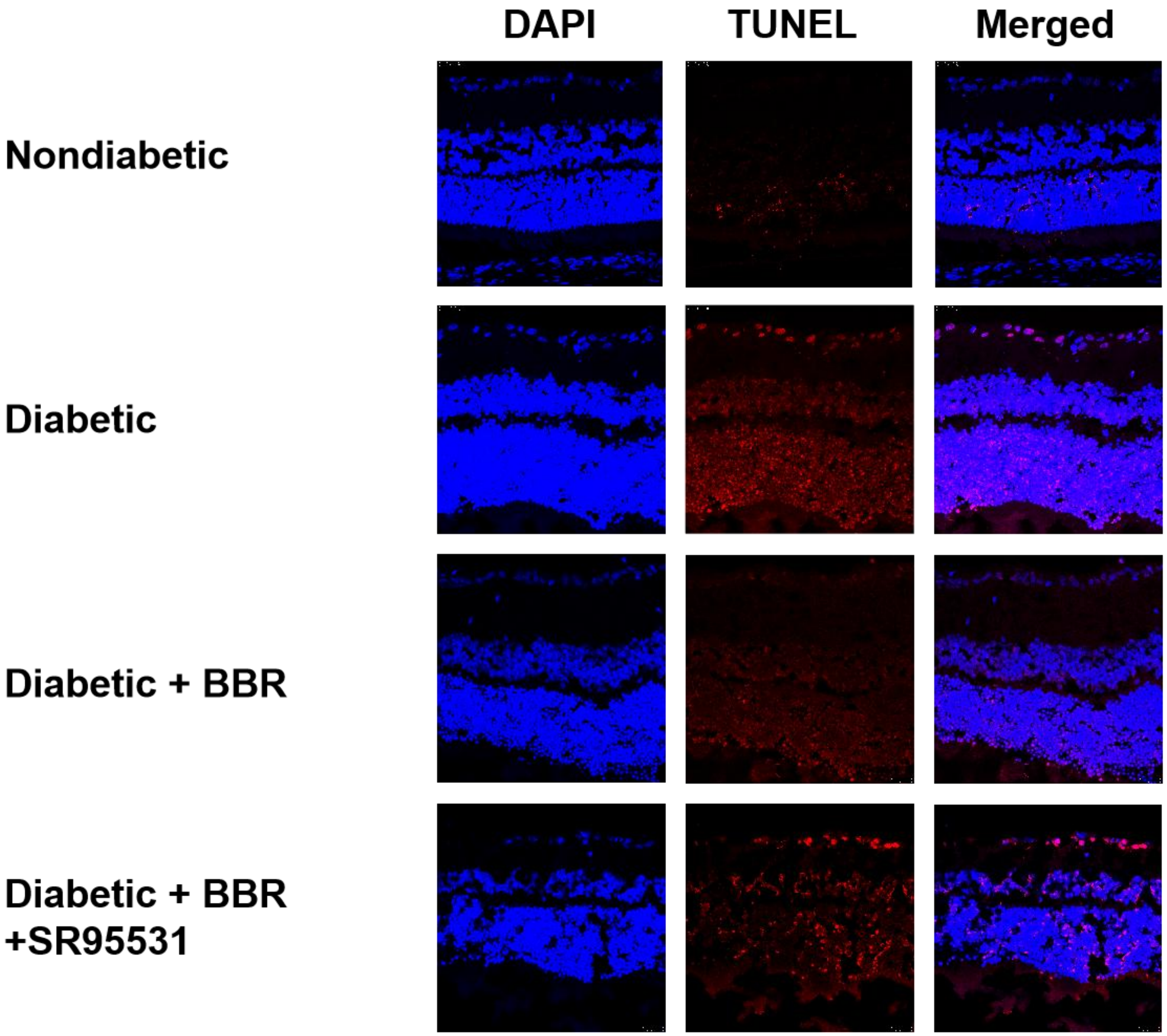

Supplementary Figure S2. Original images of TUNEL staining of **Figure 4**.

(scale bar, 25 µm)

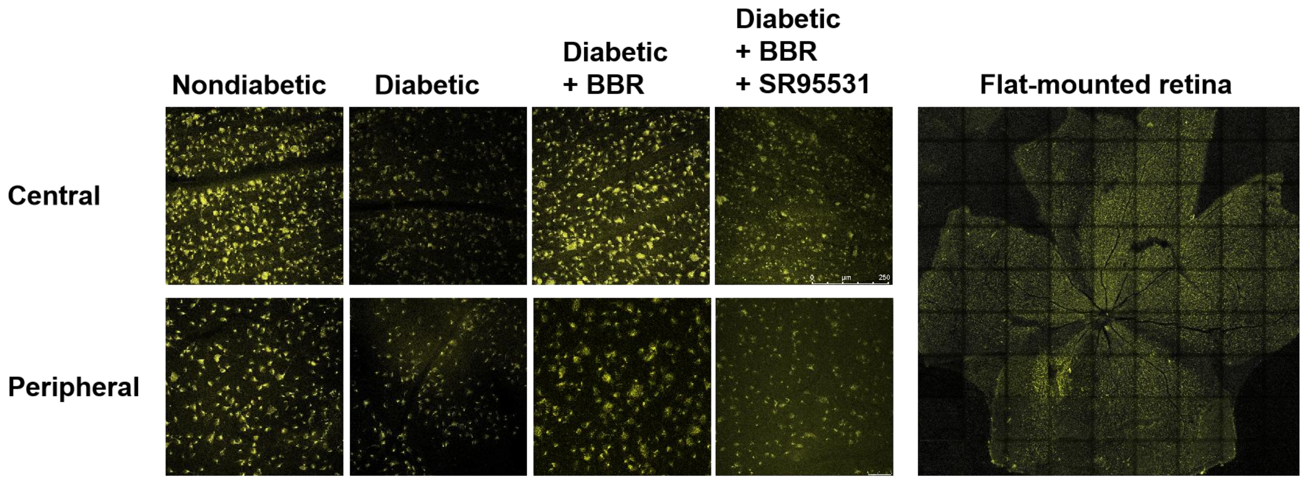

Supplementary Figure S3. Original images of FluoroGold-labeled surviving RGCs in the central and peripheral regions of flat-mounted retinas of **Figure 5**.

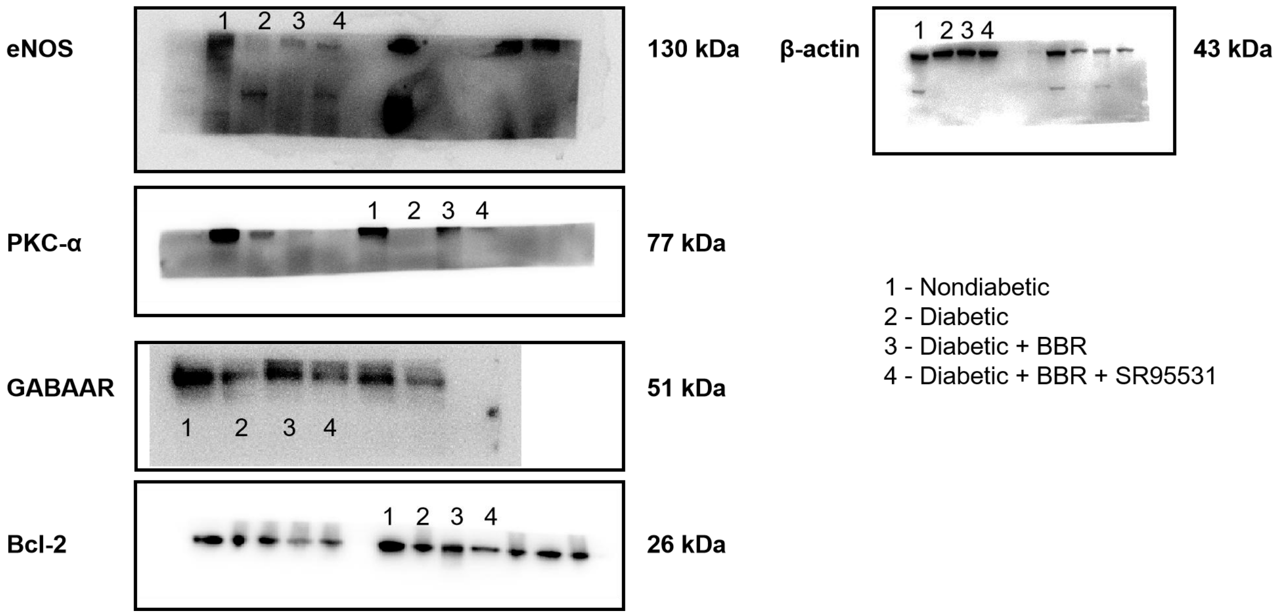

Supplementary Figure S4. Full-length blots of **Figure 9A**. Western blot showing the protein expression of eNOS, PKC-α, GABAAR, Bcl-2 and β-actin subunit in the nondiabetic, diabetic, diabetic + BBR, diabetic + BBR + SR95531 all four groups.
